# Characterization, Stability, and Bioaccessibility of Betalain and Phenolic Compounds from *Opuntia stricta var. Dillenii* Fruits and Products of Their Industrialization

**DOI:** 10.3390/foods10071593

**Published:** 2021-07-09

**Authors:** Iván Gómez-López, Gloria Lobo-Rodrigo, María P. Portillo, M. Pilar Cano

**Affiliations:** 1 Laboratory of Phytochemistry and Plant Food Functionality, Biotechnology and Food Microbiology Department, Institute of Food Science Research (CIAL) (CSIC-UAM), Nicolás Cabrera 9, 28049 Madrid, Spain; ivan.gomez@ehu.eus; 2 Nutrition and Obesity Group, Department of Nutrition and Food Science, Faculty of Pharmacy, University of the Basque Country (UPV/EHU), Lucio Lascaray Research Center, 01006 Vitoria-Gasteiz, Spain; mariapuy.portillo@ehu.eus; 3 Department of Crop Production in Tropical and Subtropical Areas, Instituto Canario de Investigaciones Agrarias (ICIA), 38270 Tenerife, Spain; globo@icia.es; 4 BIOARABA Institute of Health, 01006 Vitoria-Gasteiz, Spain; 5 CIBERobn Physiopathology of Obesity and Nutrition, Institute of Health Carlos III (ISCIII), 01006 Vitoria-Gasteiz, Spain

**Keywords:** *Opuntia stricta var. Dillenii*, fruit tissues, by-products, fruit jam, betalains, phenolic compounds, in vitro gastrointestinal digestion, stability, bioaccessibility

## Abstract

The aim of the present study was the full characterization, quantification, and determination of the digestive stability and bioaccessibility of individual betalain and phenolic compounds of *Opuntia stricta, var. Dillenii* fresh fruits (peel, pulp, and whole fruit) and of the products of the industrialization to obtain jam (raw pressed juice (product used for jam formulation), by-product (bagasse), and frozen whole fruit (starting material for jam production)). *Opuntia stricta var. Dillenii* fruits and products profile showed 60 betalain and phenolic compounds that were identified and quantified by HPLC-DAD-ESI/MS and HPLC-DAD-MS/QTOF, being 25 phenolic acids (including isomers and derivatives), 12 flavonoids (including glycosides), 3 ellagic acids (including glycosides and derivative), and 20 betanins (including degradation compounds). In vitro gastrointestinal digestion was performed by INFOGEST^®^ protocol. Fruit pulp showed the greater content of total betalains (444.77 mg/100 g f.w.), and jam only showed very low amounts of two betanin degradation compounds, Cyclo-dopa-5-O-β-glucoside (and its isomer) (0.63 mg/100 f.w.), and two Phyllocactin derivatives (1.04 mg/100 g f.w.). Meanwhile, fruit peel was the richer tissue in total phenolic acids (273.42 mg/100 g f.w.), mainly in piscidic acid content and total flavonoids (7.39 mg/100 g f.w.), isorhamnetin glucoxyl-rhamnosyl-pentoside (IG2) being the most abundant of these compounds. The stability of betalains and phenolic compounds during *in vitro* gastrointestinal digestion is reported in the present study. In *Opuntia stricta var. Dillenii* pulp (the edible fraction of the fresh fruit), the betanin bioaccessibility was only 22.9%, and the flavonoid bioaccessibility ranged from 53.7% to 30.6%, depending on the compound. In non-edible samples, such as peel sample (PE), the betanin bioaccessibility was 42.5% and the greater bioaccessibility in flavonoids was observed for quercetin glycoside (QG1) 53.7%, the fruit peel being the most interesting material to obtain antioxidant extracts, attending to its composition on antioxidant compounds and their bioaccessibilities.

## 1. Introduction

Prickly pear (*Opuntia* spp. L Mill) fruits belong to the most abundant genus within the *Cactaceae* family [1]. This dicotyledonous angiosperm plant was one of the first species introduced to Europe from the New World by the Spanish conquerors in 1548–1570 [2]. Due to its remarkable adaptation to arid and semiarid climates, it has been spread to many subtropical areas, such as the Mediterranean coast, China, South Africa, and Australia [3,4].

Initially, the prickly pear was brought to Europe as a feeding base for carmine cochineal (*Dactylopius coccus* Costa) in order to produce colorant for the industry. However, prickly pear fruits contain a high content of important nutrients (organic acids, lipids, minerals, vitamins, etc.) and bioactive compounds, such as polysaccharides, betalains (betacyanins and betaxanthins), and phenolic compounds such as phenolic acids (piscidic acid) and flavonoids (isorhamnetin glycosides), with proven biological activities [5,6,7]. For the nutritional, health, and agronomical potential that they have, some studies have remarked upon the importance of exploiting them as food products [8]. For this reason, the food industry is starting to produce prickly pear products such as jam and juices, regarding consumers’ new habits of looking for health-promoting processed food products.

In Spain, recent reports showed that 875 t per year of *Opuntia ficus-indica* fruits was cultivated. The main production of this fruit takes place in the Canary Islands (535 t in 2018) [9]. This fruit is seasonal, and it can only be harvested from June to September (in the northern hemisphere). However, due to the Canary Islands’ climate, the *Opuntia* fruits are available until December [2,10]. *Opuntia* spp. contains about nearly 300 species; despite this, *Opuntia ficus-indica* is the most extensive, consumed, and investigated fruit [1,8,11]. In the Canary Islands, there are other interesting varieties growing wildly around all islands, such as the *Opuntia stricta var. Dillenii*, about which few scientific works have been published. For this reason, *Opuntia stricta var. Dillenii* fruits and their derived products of the jam manufacture were selected for the present study.

**Figure 1:**
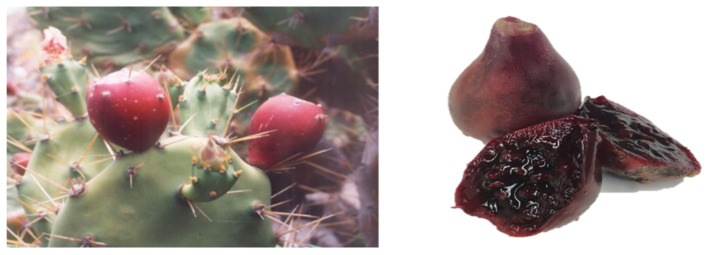
*Opuntia stricta var. Dillenii* cactus and cactus from Canary Islands, Spain.

The *Opuntia stricta var. Dillenii* fruit is a small haw, and it is formed by a deep red-purple thick peel and a red-purple pulp with abundant small seeds (Figure 1). The main compounds responsible for the purple peel and pulp color of the *Opuntia stricta var. Dillenii* fruit tissues are betalains, nitrogen-based pigments. The predominated betalain compound in *Opuntia stricta var. Dillenii* fruit is a betacyanin (purple color compound of the betalain group), specifically betanin [12]. Betalains can play a role as antioxidants by acting as radical scavengers, improving the body’s redox balance, and decreasing lipid oxidation and have a hepatoprotective and gene expression modulation [6,13,14]. *Opuntia* fruits are also a good source of phenolic compounds such as phenolic acids, piscidic acid and flavonoids, and isorhamentins that are found in their glycosylated form. Previous studies reported that the isorhamentin 3-O-rutinoside is the dominant flavonoid compound in this *Opuntia stricta var. Dillenii* [8]. Isorhamentin flavonoids play a role in the suppression of the PRAPγ gen activity, helping in the adipogenesis and slimming down, improving insulin resistance, and reducing the hepatic-esteatosis in diet-induced obese rats [13]. In addition, other phenolic compounds also have chemiprotective effects due to their anti-inflammatory effect [6]. Some studies have been reported that prickly pear fruits such as *Opuntia ficus-indica* have considerable antioxidant, anti-inflammatory, and anti-carcinogenic effects and they have suitable properties for the treatment of obesity and diabetes diseases [5,13,14,15]. However, regarding *Opuntia stricta var. Dillenii*, there are only a few studies reporting its promising biological and pharmacological properties, summarized in a recent published review [15].

Nevertheless, for antioxidants to bring the mentioned health benefits, the bioactive compounds must remain stable during the digestion process until the gastrointestinal tract in order to be absorbed and arrive at the target tissue. The problem is that the *Opuntia* fruit bioactive compounds (mainly betalains and also phenolic compounds) are very unstable under different conditions such as pH, light, temperature, and presence of oxygen. Some of these factors can produce many changes in the chemical structure of bioactive compounds during gastrointestinal digestion, producing the loss of their potential health benefits and decreasing their absorption by epithelial cells. This makes it difficult to know the percentage of the initial compound that arrives at the target tissue [10,16]. In order to determine the quantity of a compound that is liberated from the fruit tissues in the gastrointestinal tract and that is accessible for absorption (bioaccessibility) [13], the fruit samples can be submitted to an in vitro simulated gastrointestinal digestion process. The INFOGEST^®^ method is the protocol used in this study to determine the digestion stability and bioaccessibility of each bioactive compound from different fruit tissues and from the by-product of *Opuntia stricta var. Dillenii*. The INFOGEST^®^ method is the result of an international consensus for the gastrointestinal digestion simulation to obtain information about the bioaccessibility and stability of food components [16].

The aim of this study was to characterize and quantify for the first time almost all the individual bioactive compounds (betalains and phenolic compounds) in different tissues and by-products (peel, pulp, whole fruit, juice, solid-by-products (bagasse), and jam) of *Opuntia stricta var. Dillenii* fruits from Lazarote island (Canary Islands, Spain). Additionally, a study of the digestive stability and bioaccessibility of these *Opuntia stricta var. Dillenii* bioactive compounds through INFOGEST^®^ in vitro gastrointestinal digestion was carried out. The digestive stability of antioxidant compounds (betalains and phenolic compounds) from different tissues of *Opuntia stricta var. Dillenii* fruit and by-products were analyzed in order to (i) determine the relevance of the edible fractions of fruits such as pulp and manufacture products such as jam and (ii) to explore the new potential of the industrial by-products (peel, bagasse, whole fruit (non-uniform and non-commercial fruits)) and intermediate product (raw juice) as new resources to obtain biologically active food ingredients.

## 2. Materials and Methods

### 2.1. Solvents, Reagents, and Standards

Ultra-pure water was obtained from a Milipak^®^ Express 40 system (Merk-Milipore, Dormstadt, Germany). Methanol (99.8% LC-MS) was acquired from VWR International (Barcelona, Spain). Formic acid was purchased from Panreac Química (Barcelona, Spain). Sephadex LH-20, standards (isorhamnetin, quercetin, rutin, gallic acid, 4-hydroxybenzoic acid), α-amylase (10080; 79 U mg/L), pepsin (P6887; 791 U mg/L), pancreatin (P7545, 17 units TAME per mg), bile (B8381), and other reagents used for the in vitro digestion assay were supplied by Sigma-Aldrich (St. Louis, MO, USA). Using a commercial betalin-rich concentrate extract of commercial beetroot, the betanin was purified by a Sephadex L20 resin, and betaxanthins were semi-synthesized using purified betanin based on protocols reported by García-Cayuela et al. (2019) [4]. Phyllocactin was isolated from cactus berry fruits (*Myrtillocactus geometrizans*) using semi-preparative high-performance liquid chromatography (HPLC) described by Montiel-Sánchez et al. (2020) [17]. With respect to phenolic compounds, piscidic acid was purified from prickly pear peels by semi-preparative high-performance liquid chromatography (HPLC), also described by García-Cayuela et al., (2019) [4]. Eucomic acid and derivatives were quantified using the the tyrosol standard [6]. Isorhamnetin glycosides standards were supplied from Dr. Serna-Saldivar’s laboratory in the Biotechnology center FEMSA (Escuela de Ingeniería y Ciencias, Instituto Tecnológico de Monterrey, Monterrey, Mexico). Other phenolic compounds such as gallic acid, ferulic acid, protocatechuic acid, p-hydroxybenzoic acid, quinic acid, ellagic acid, p-coumaric acid, quercetin, myricetin, rutin, and kaempferol standards were purchased from Sigma-Aldrich (St. Louis, MO, USA).

### 2.2. Plant Material

**Figure 2:**
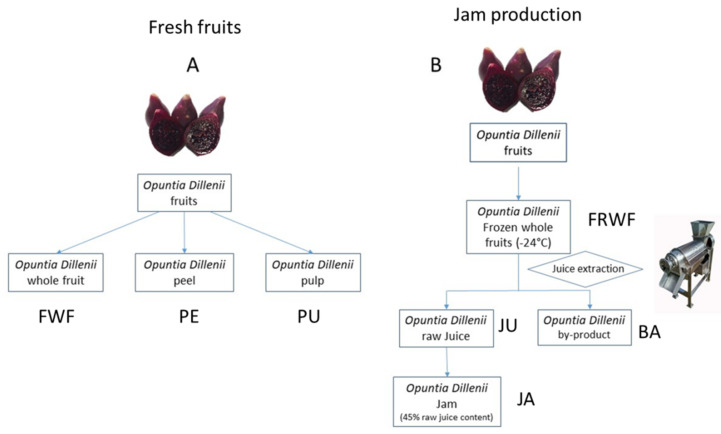
Scheme of *Opuntia stricta var. Dillenii* samples. (**A**) Fresh fruit samples (whole fruit FWF, peel PE, pulp PU), and (**B**) samples from jam production: frozen whole fruits, FRWF; raw juice, JU; by-product (bagasse), BA; jam, JA.

*Opuntia stricta var. Dilleni* fruits were collected in Tinajo (Lanzarote, Canary Islands, Spain; 29°3′ N, 13°4′ W; 209 m over sea level) in July of 2019. Mature fruits with no physical damages were selected (Figure 1). Selected fruits were washed, and their tissues were manually processed into peels, pulps, and whole fruits. Jam from *Opuntia stricta var. Dilleni* fruits was supplied by a Spanish company, Bernardo’s (Lanzarote, Spain). Additionally, this company supplied the starting material (slowly frozen whole fruits) and intermediate product (raw juice), together with the by-product, a mash named bagasse. Figure 2 shows the different *Opuntia stricta var. Dillenii* materials studied in the present work. In the company plant, *Opuntia* fruits were immediately stored in a cabin room at −24 °C, slowly freezing the fruits. Raw juice (JU) was produced from the frozen whole fruits (FRFW) by a squeezing process, and this product (JU) was the starting material to make the *Opuntia stricta var. Dillenii* jam (JA) (Figure 2). In the present study, fresh fruits (whole fruit (FWF), peel (PE), and pulp (PU)) and frozen whole fruits (FRWR), raw juice (JU), jam (JA, containing 45% of raw juice), and the by-product (bagasse (BA)) were studied. Figure 2 shows the scheme of the *Opuntia stricta var. Dillenii* studied in the present work.

After receiving the samples in the research center in Madrid (Spain), the *Opuntia stricta var. Dillenii* fruits were washed and selected according to similar skin coloration and size, free of damages. Table S1 (Supplementary Materials) shows the physicochemical characteristics of these fresh fruits. The analysis of the physicochemical characteristics of the fresh whole fruits was carried out using 15 fruits, and the rest of *Opuntia* fruits were divided into fruits that were sliced into cubes (1 × 1 cm), which were the whole fresh fruit samples (FWF), and rest of the fruits that were manually separated into peels (PE) (endocarp and exocarp) and pulps (PU) (mesocarp), Figure 2. All these samples were immediately frozen with liquid nitrogen to stabilize them until analysis. Additionally, industrial *Opuntia* samples (slowly frozen whole fruits (FRWF), juice (JU), jam (JA), and the by-product (BA) were also frozen with liquid nitrogen. All frozen by liquid nitrogen *Opuntia* samples were freeze-dried for 5 days at −45 °C and 1.3 × 10^−3^ MPa (LyoBeta 15, Azbil Telstar, S.L., Terrasa, Spain) and pulverized in a knife mill (Grindomix GM200, Retsch, Germany) to a fine particle size (<2 mm) and seeds were removed in pulps. Samples were vacuum-sealed and stored at −24 °C until analysis.

### 2.3. Physicochemical Analysis

The physicochemical characteristics such as apical caliber (cm), equatorial caliber (cm), weight (g), firmness (N), and peel, pulp, and seed proportion (%) were determined directly in fifteen (15) whole fruits (Supplementary Materials Table S1). Titratable acidity (g citric acid/100 g fresh weight) was analyzed by neutralization of *Opuntia* whole fruit pear juice with 0.1 N sodium hydroxide until a pH value of 8.1. pH and soluble solids (°Brix at 25 °C) were also measured from juice obtained from prickly pear pulps. The color of peels and pulps was recorded using the L* (lightness), a* (green-red tonality), and b* (blue-yellow tonality) scale CIELAB system with a Konica Minolta CM-3500d (Japan).

### 2.4. Betalain and Phenolic Compounds Extraction for Characterization

For characterization of betalains and phenolic compounds, extracts were obtained from all freeze-dried and pulverized samples under diminished light, as reported previously Gómez-Maqueo et al. [10]. Freeze-dried *Opuntia stricta var. Dillenii* samples were extracted with 5 mL methanol:water (1:1, *v*:*v*), and the extraction process was repeated two more times. The last extraction was made with 3 mL of pure methanol, and the combined supernatants were dried with a vacuum. Aqueous extracts were filtered (0.45 µm nylon filter (E0032, Análisis Vínicos, Spain) and analyzed by HPLC.

### 2.5. In Vitro Digestion Assay

The *in vitro* gastrointestinal digestion assay was performed according to the standardized INFOGEST protocol [16,17], using rehydrated freeze-dried samples, except jam that is extracted directly. The solutions for mouth (Simulated Saliva Fluid, SSF), stomach (Simulated Gastric Fluid, SGF), and small-intestinal (Simulated Duodenal Fluid, SDF) compartments were prepared according to a previous article [18]. The addition of enzymes in the preparation of digestive fluids was performed daily and moments prior to the digestive assay. After each phase (oral, gastric, and intestinal) of the simulated digestion, samples were frozen with liquid N_2_ and stored at −24 °C.

After obtaining all phases of the *in vitro* gastrointestinal digestion, digestive phases were thawed, and extracts containing the betalains and phenolic compounds were obtained following the method previously reported by Gómez-Maqueo et al. [10]. After the extraction process, the aqueous samples were filtered with a 0.45 µm syringe filter into a vial and analyzed by HPLC.

The bioaccessibility of *Opuntia stricta var. Dillenii* betalains and phenolic compounds was calculated as the ratio between their concentration in the intestinal fraction and their initial concentration in the fruit (Equation (1)) [10].

(1) $$Bioaccessibility\;\left(\%\right)=\frac{concentration\;in\;the\;intestinal\;fraction\;}{initial\;concentration\;in\;the\;fruit\;}\times 10$$

### 2.6. Betalain and Phenolic Characterization and Quantification by High-Performance Liquid Chromatography

Betalains and phenolic compounds were determined simultaneously by high-performance liquid chromatography, as reported previously by our research team [4,10,17]. A 1200 Series Agilent HPLC System (Agilent Technologies, Santa Clara, CA, USA) with a reverse-phase C18 column (Zorbax SB-C18, 250 × 4.6 mm i.d., S-5 µm; Agilent) at 25 °C was used. Mobile phase A was 1% formic acid (*v/v*) in ultrapure water, and mobile phase B was 1% formic acid (*v/v*) in methanol. Separation was achieved using an initial composition of 15% B during 15 min, increased to 25% within 10 min, subsequently ramped to 50% B within 10 min, increased to 75% B in 15 min, and finally followed by a decrease period of 15% B in 5 min prior to isocratic re-equilibration for 10 min. The flow rate was 0.8 mL/min, and the injection volume was 20 µL. The UV-visible photodiode array detector was set at four wavelengths to detect phenolic acids (280 nm), flavonoids (370 nm), betaxanthins (480 nm), and betacyanins (535 nm). U*V/V*is spectra were additionally recorded between 200 and 700 nm. The HPLC-DAD was coupled to a mass spectrometry detector (LCMS SQ 6120, Agilent) with an electrospray ionization (ESI) source operating in positive ion mode. The drying gas was nitrogen at 3L/min at 137.9 KPa. The nebulizer temperature was 300 °C, and the capillary had 3500 V potential. The coliseum gas was helium, and the fragmentation amplitude was 70 V. Spectra were recorded *m/z* from 100 to 1000.

Further mass spectrometry analyses were performed in a maXis II LC-QTOF equipment (Bruker Daltonics, Bremen, Germany) with an ESI source and the same chromatographic conditions. The ESI-QTOF detector worked in positive ion mode and recorded spectra *m/z* from 50 to 3000. Operation conditions were 300 °C, 3500 V capillary voltage, 2000 V charging voltage, 2.0 bar nebulizer, and dry gas at 6 L/ min. MS/MS analysis used the bbCID (broad-band collision induces dissociation) method at 30 eV.

Compounds were identified according to their retention times, U*V/V*is, and mass spectral data compared to those of commercial, semi-synthesized, or purified standards. Quantitation of most betalains, piscidic acid, rutin, isorhamnetin glycosides, and kaempherol glycoside was determined using the calibration curves of the corresponding isolated standards. Eucomic acid and derivatives were quantified using tyrosol standard [6]. Quercetin glycosides were quantified by using the rutin calibration curve. The identification of betalains and phenolic compounds in *Opuntia stricta var. Dillenii* samples are shown in Table 1.

### 2.7. Statistical Analyses

All statistical analyses were conducted by means of the SPSS^®^ Statistics version 26.0 software for Windows (IBM Corp, Armonk, NY, USA). Results were represented as mean ± standard deviation (*n* = 2). Results obtained for the variables studied in the different groups were compared by one-way analysis of variance (ANOVA) with significant differences (*p* < 0.05) using a Tukey-b test.

## 3. Results

### 3.1. Physicochemical Characteristics of Opuntia stricta, var. Dillenii Whole Fruit

All physicochemical characteristics are presented in the Supplementary Materials (Table S1). *Opuntia stricta, var. Dillenii* fruit was characterized by small size (6.10 ± 0.60 cm apical caliber and 3.96 ± 0.14 cm equatorial caliber), low weight (54.35 ± 6.82 g), and a dark purple color, which was evaluated by CIELAB, being the pulp color (30.92 ± 1.39 L*, 4.47 ± 3.26 a*, (−7.46) ± 0.51 b*) and peel color (29.03 ± 0.67 L*, 4.07 ± 0.69 a*, (−8.09) ± 0.91 b*). According to the statistical analysis, no significant differences were found between the peel and pulp color. Additionally, many authors reported a large number of seeds in this cactus fruit [1,5]; in the present study, the percent of seeds was 32.80 ± 0.46 of the weight of whole fruit. *Opuntia stricta var. Dillenii* fresh whole fruit (FWF) showed a pH of 3.55 ± 0.08, a titratable acidity of 1.58 ± 0.10 (g citric acid/100 g f.w.), and soluble solids of 10.80 ± 0.30 ºBrix. According to other authors [1,8], *Opuntia stricta, var. Dillenii* has lower soluble solids (Brix) and pH and higher titratable acidity than other *Opuntia* fruits, i.e., *Opuntia ficus-indica* fruits, as reported by Gómez-Maqueo et al. [10].

### 3.2. Identification of Betalains and Phenolic Compounds

#### 3.2.1. Betalains

**Figure 3:**
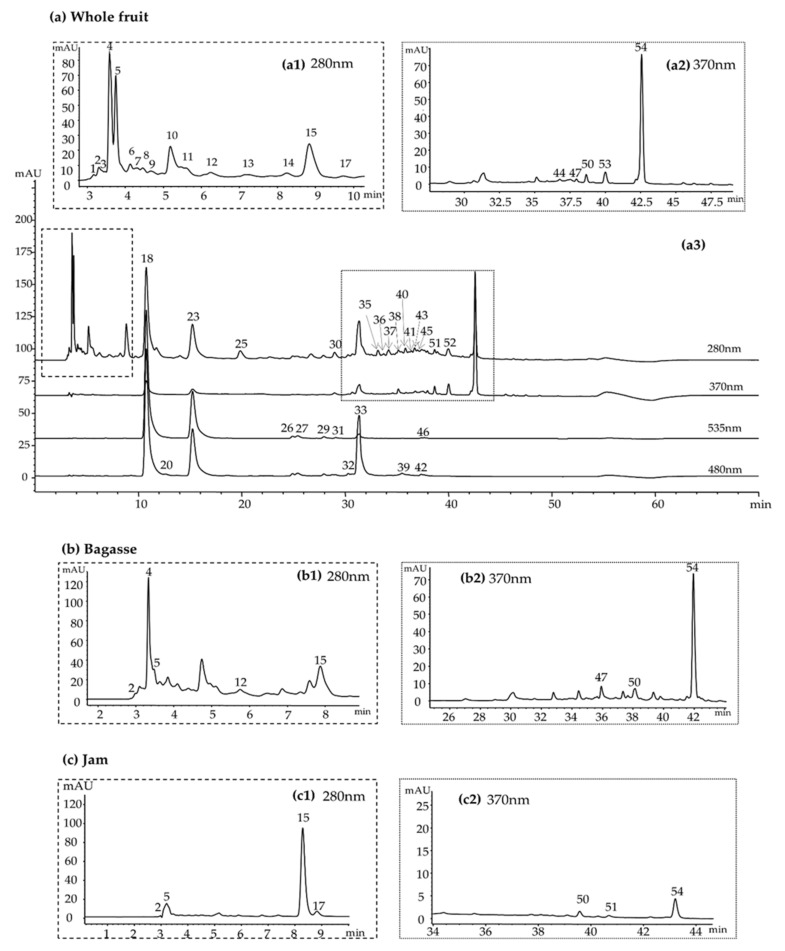
HPLC-DAD chromatogram of betalains and phenolic compounds in *Opuntia stricta var. Dillenii* from (**a**) whole fruit at 280 nm (zoom chromatogram 0–10 min, **a1**), 370 nm (zoom chromatogram 25.5–50 min, **a2**), and all chromatograms with UV-vis detection: 280, 370, 480, and 535 nm (**a3**), (**b**) by-product (bagasse) from the industrialization, chromatogram at 280 nm (zoom chromatogram 0–10 min, **b1**) and at 370 nm (zoom chromatogram 25.5–50 min, **b2**) (**c**) and *Opuntia stricta var. Dillenii‘s* jam chromatograms at 280 nm (zoom chromatogram 0–10 min, **c1**) and at 370 nm (zoom chromatogram 25.5–50 min, **c2**). Numbers correspond to the identified compounds indicated in Table 1.

The bioactive compound profile of *Opuntia stricta var. Dillenii* fruits and by-products was analyzed using HPLC-DAD and HPLC-ESI/MS (with ESI and QTof detectors) UV-vis detection. Figure 3 shows the chromatograms obtained for fresh whole fruit (FWF), by-product (mash) of jam production (BA), and commercial jam (JA). Retention times (Rt), U*V/V*is spectra, and MS spectral data of the identified individual compounds are shown in Table 1 for all *Opuntia stricta var. Dillenii* samples studied in the present work.

In the fresh whole fruit (FWF) chromatograms, detected at 535 and 480 nm, 12 betalain compounds were identified, and betanin, Peak 18 (Rt = 10.76min; λ_max_ 535 nm), Mw 550, and MS/MS (*m*/*z*) fragments (390, 389) were the most important betalain compounds in the *Opuntia stricta var. Dillenii* whole fruit. Among the identified betalains, five of them that had a maximum absorption at 430 nm were identified as neobetanins. Peak 33 (Rt = 31.34 min; λ_max_ 468 nm) was identified as a neobetanin with Mw 548 and MS/MS (*m/z*) fragment (387). *Opuntia stricta var. Dillenii* fresh whole fruit (FWF) and tissues (peel (PE) and pulp (PU)) from Canary Islands (Spain) did not show the presence of indicaxanthin or other betaxanthin compounds. In the present study, other betacyanins were also identified such as 17-decarboxy-betanin (Peak 20, Rt = 11.73 min and λ_max_ 504 nm), isobetanin (Peak 23, Rt = 15.23 min and λmax 535 nm), betanidin (Peak 26, Rt = 24.91 min and λ_max_ 538 nm), and 17-descarboxy-Neobetanin (Peak 32, Rt = 30.27 min and λmax 451nm) in *Opuntia stricta var. Dillenii* samples, together with other betalains such as 6’-O-sinapoyl-O-Gompherin (betanidin-6-O-sinapoylglucoside) (Peak 27, Rt = 25.42 min and λ_max_ 539 nm), 2′-O-apiosyl-4-O-Phyllocactin (Peak 29, Rt = 27.90 min and λ_max_ 537 nm), and 5″-O-E-sinapoyl-2′-apyosil-Phyllocactin (Peak 31, Rt = 29.08 min and λ_max_ 540 nm) (Table 1).

*Opuntia stricta var. Dillenii***jam sample** (JA) obtained from the standard thermal processing to obtain jams showed the presence of other betalain compounds produced by thermal degradation of betanin such as cyclo-Dopa-5-O-β-glucoside, Peak 21 (Rt = 12.97 min; λ_max_ 358 nm) with Mw 357 and MS/MS (*m/z*) fragment (196) and cyclo-Dopa-5-O-α-glucoside (isomer), and Peak 22 (Rt = 14.42min; λ_max_ 358 nm) with Mw 357 and MS/MS (*m/z*) fragment (196) (Figure 3c). Meanwhile, *Opuntia stricta* **by-product** (BA), obtained from the first stage of jam processing (Figure 2), showed a similar bioactive profile to the **fresh whole fruit** sample (FWF) (Figure 3b and Table 1).

#### 3.2.2. Phenolic Compounds

*Opuntia stricta var. Dillenii* sample extracts showed the presence of thirty-two (32) phenolic compounds by HPLC recorded at λmax 280 nm (phenolic acids) and 370 nm (flavonoids). Figure 3 showed the chromatograms obtained at 280 and 370 nm for phenolic compound characterization in **whole fresh fruit** (FRF) in Figure 3a, **by-product** bagasse (BA) in Figure 3b, and **jam** (JA) in Figure 3c. Piscidic acid, Peak 15 (Rt = 8.83 min; λ_max_ 272 nm) with Mw 254 and MS/MS (*m/z*) fragments (193, 165, 135, 119, 107), was the most remarkable compound among the phenolics acids identified in all *Opuntia stricta var. Dillenii* samples. Additionally, isomers such as piscidic acid isomer I and II, Peaks 4 and 5, with the same Mw 254, were identified. Peak 25 (Rt = 26.70 min; λ_max_ 278 nm) was identified as eucomic acid, another important phenolic acid in *Cactaceae* family. In the present work, eucomic acid isomers I and II (Peak 17 and 28, respectively) and a eucomic acid derivative (Peak 14) were also identified in fresh fruit samples (**whole fruit, peel and pulp**) of *Opuntia stricta var. Dillenii* (Table 1). Other phenolic acids were identified in *Opuntia dillenii* samples such as Ferulic acid (Peak 36) and two of their derivatives (Peaks 7, 9), together with p-Hydroxybenzoic acid (Peak 13) and some glycosides such as 4-Hydroxybenzoic acid 4-O-glucoside (Peak 35) and its isomer, 4-Hydroxybenzoic acid 4-O-glucoside isomer (Peak 37), Table 1. In addition, Protocatechuic acid derivative (Peak 10) with Mw 296 and MS/MS fragments (286, 153, 86) was tentatively identified in *Opuntia dillenii* **fresh whole fruit** (FWF) and **fresh pulp** (PU) samples.

With respect to flavonoids, six (6) Isorhamentin glycosides (IG1, IG2, IG3, IG4, and Isorhamentin glucoside), three (3) quercetin glycosides (QG1, QG2, and Rutin (quercetin-3-rutinoside)), one (1) Myricetin glycoside (myricitrin) (Peak 51), and one (1) Kaempferol glycoside (KG1) (Peak 58) were identified in all *Opuntia stricta var. Dillenii* samples (Table 1). Additionally, ellagic acid (Peak 47) and some of their derivatives were detected, mainly in **fresh whole fruit** samples (FWF), ellagic acid rhamnoside (Peak 48, λ*max* 352 nm) being the identified ellagic acid compound found in almost all *Opuntia stricta var. Dillenii* samples. All their spectral characteristics are shown in Table 1.

### 3.3. Quantification of Betalains and Phenolic Compounds

#### 3.3.1. Quantification of Betalains

**Figure 4:**
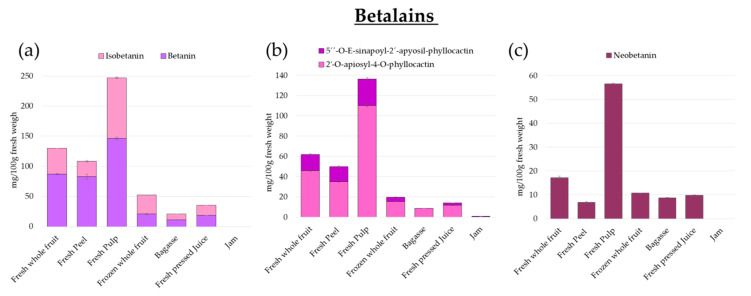
Betalains content (mg/100 g fresh weight) in *Opuntia stricta var. Dillenii* fruit tissues (peel, pulp, and whole fruit), jam production products (intermediate juice and jam), and by-product (bagasse); (**a**) betanin and isobetanin; (**b**) 2′-O-apiosyl-4-O-phyllocactin and 5″-O-E-sinapoyl-2′-apyosil-phyllocactin; (**c**) neobentanin.

Individual content in betalain compounds in the *Opuntia stricta var. Dillenii* samples from the Canary Islands is presented in Table S2, Supplementary Materials. **Fresh whole fruit** (FWF) and **fresh pulp** (PU) showed the highest content in betalains, 47% and 58%, respectively, **jam** (JA) being the sample with a lower betalain total content (1%) due to the thermal degradation of these compounds that took place in the jam production process (Figure 4 and Figure S1, Supplementary Materials). The most abundant betalain compound in all *Opuntia stricta var. Dillenii* samples was betanin, **fresh pulp** of the *Opuntia dillenii* being the sample with the highest content in this betalain, 146.36 ± 2.08 mg/100 g f.w. (Figure 4 and Table S2, Supplementary Materials). Additionally, the content of other betacyanins such as isobetanin was higher in **fresh pulp** (100.77 ± 1.48f mg/100 g f.w.) and the **fresh whole fruit** sample (42.78 ± 0.04 mg/100 g f.w.) (Figure 4). The **by-product** (BA) sample, the fruit mash residue from the juice production, had a 1.5-fold lower total content of betacyanins than the content found in raw **juice** (JU) (Figure 4). In the present study, *Opuntia stricta var. Dillenii* raw **juice** (JU), which was obtained from **frozen whole fruit** (FRFW), showed a nearly 10% loss of betanin content (Table S2); meanwhile, in this sample, an important increase in the content of isobetanin (31.42 ± 0.09 g f.w.), a C15 epimer of betanin produced by the isomerization of betanin, was observed. Additionally, *Opuntia dilenii* raw **juice** (JU) and **by-product** (BA) contained other betanin degradation compounds in different amounts, as 17-Decarboxy-betanin, 17-Decarboxy-isobetanin, betanidin, and 17-Descarboxy-neobetanin (Table S2, Supplementary Materials). Anther betacyanin, 2′-O-apiosyl-4-O-Phyllocactin, also appeared in these processing samples, JU and BA, in low amounts (Figure 4).

Neobetanin content *Opuntia dillenii* pulp (PU) was the highest among all analyzed samples (Figure 4c and Table S2). Other samples from processing as raw juice (JU) and by-product (BA) showed similar content of neobetanin, Table S2 (Supplementary Materials). In contrast, *Opuntia stricta var. Dillenii* **jam** (JA) only showed very low amounts of total betalains (1.67 ± 0.08 mg/100 g f.w.), Cyclo-dopa-5-O-β-glucoside (0.57 ± 0.01 mg/100 g f.w.) and its isomer being the most abundant betanin compounds, produced by the thermal degradation of betanin. Additionally, very low amounts of 2′-O-apiosyl-4-O-Phyllocactin (0.51 ± 0.03 mg/100 g f.w.) and 5″-O-E-sinapoyl-2′-apyosil-Phyllocactin (0.53 ± 0.03 mg/100 g f.w.) were quantified in **jam** (JA), (Figure 3b and Table S2, Supplementary Materials).

#### 3.3.2. Quantification of Phenolic Compounds

**Figure 5:**
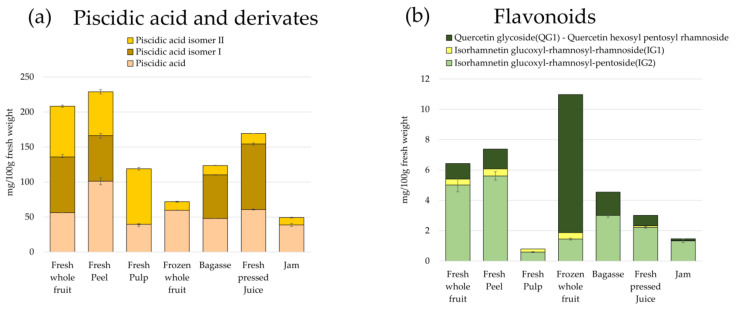
Phenolic compounds content (mg/100 g fresh weight) in *Opuntia stricta var. Dillenii* fruit tissues (peel, pulp, and whole fruit), jam production products (intermediate juice and jam), and by-product (bagasse) (**a**) phenolic acids: piscidic acid, piscidic acid isomer I, and piscidic acid isomer II; (**b**) flavonoids: quercetin glycoside(QG1)—quercetin hexosyl pentosyl rhamnoside, isorhamnetin glucoxyl-rhamnosyl-rhamnoside(IG1), and isorhamnetin glucoxyl-rhamnosyl-pentoside (IG2).

As other studies reported about *Opuntia ficus-indica* fruits and tissues [13], *Opuntia stricta var. Dillenii* **peel** (PE) had the highest content of total phenolic compounds (as the sum of individual phenolic compound content), 273.42 ± 13.67 mg/100 g f.w., among all samples studied in the present work (Table S2, Supplementary Materials). The most abundant phenolic compounds in *Opuntia stricta var. Dillenii* samples were piscidic acid (56.21 ± 0.30c mg/100 g f.w.) and its isomers I (79.65 ± 3.66 mg/100 g f.w.) and II (72.54 ± 1.54 mg/100 g f.w.). These phenolic acids were also more abundant in **whole fresh fruit** (FWF) and **peel** (PE). Figure 5 (a) and Table S2 (Supplementary Materials) showed the content in these phenolic acids in the studied *Opuntia dillenii* samples. Ferulic acid (and derivatives) content was also higher in **fresh whole fruit** (FWF) with a total amount of 2.49 mg/100 g f.w.; meanwhile, in **frozen whole fruit** (FRWF), only a Ferulic acid derivative III can be quantified (0.97 ± 0.04 mg/100 f.w.). With respect to the eucomic acid and its isomers (and derivative), the highest amount was found in the fruit **pulp** (PU) with a 14.61 ± 0.56 mg/100 g f.w., and the lowest content was found in **jam** (JA) (1.46 ± 0.03 mg/100 g/100 g f.w. (as the eucomic isomer I), Table S2 (Supplementary Materials). In addition, different amounts of other phenolic acids such as Gallic acid and its derivative (1.13 mg/100 g f.w.); Quinic acid (7.81 ± 0.69 mg/100 g f.w.); p-Hydroxybenzoic acid and its glucoside, including the isomer (3.24 mg/100 g f.w.); Protocatechuic acid derivative I and II (5.13 mg/100 g f.w.); and p-Coumaric acid and its derivative (0.95 mg/100 g f.w.) were quantified in **fresh whole fruit** (FWF), Table S2 (Supplementary Materials).

Flavonoid compounds are also present in *Opuntia stricta var. Dillenii* in different amounts depending on the analyzed sample (Figure 5 and Table S2, Supplementary Materials). Total flavonoid content, as the sum of individual flavonoid content quantified by HPLC, ranged from 12.17 mg/100 g f.w. in the **by-product** (BA) sample to 0.79 mg/100 g f.w in fruit **pulp** (PU). In the present study about *Opuntia stricta var. Dillenii*, the most abundant flavonoid in **fresh whole fruit** (FWF) was isorhamnetin glucoxyl-rhamnosyl-pentoside (IG2) (5.01 mg/100 g f.w.), followed by quercetin glycoside (QG1, quercetin hexosyl pentosyl rhamnoside) (1.02 mg/100 g f.w.) and isorhamnetin glucoxyl-rhamnosyl-rhamnoside (IG1) (0.40 mg/100 g f.w.) (Figure 5b). In contrast to *Opuntia ficus-indica*, the fruits of *O. Dillenii* showed higher amounts of flavonoids in fruit **peel** (PE) (7.39 mg/100 g f.w.) than in **pulp** (PU) (0.79 ± 0.04 mg/100 g f.w.), Table S2 (Supplementary Materials). *O. Dillenii* samples from jam processing also showed low amounts of flavonoids, quercetin glycoside (QG1) being the most abundant compound (Figure 5b) (9.11 ± 0.17 mg/100 g f.w.) in **frozen whole fruit** (FRWF), the starting material to obtain jam (Figure 2); however, losses of total flavonoid content of nearly 45% in the **by-product** (BA), a loss of 75% in raw **juice** (JU), and a loss of 88% in the final product, the *Opuntia Dillenii* **jam** (JA), were observed (Figure 5b and Table S2, Supplementary Materials).

### 3.4. Stability and Recovery of Opuntia stricta var. Dillenii betalains and Phenolic Compounds during In Vitro Gastrointestinal Digestion

The description of the *in vitro* gastrointestinal digestion assay data and the discussion of the obtained results will be undertaken attending to the characteristics of the studied samples of *Opuntia stricta var. Dillenii* in the present work. First, we will discuss the results of the stability of the antioxidant bioactives in edible samples such as *Opuntia* fruit **pulp** (PU) and **jam** (JA), and after that, the bioactive stability data in the *Opuntia* fruit processing to obtain fruit jam, the starting material (**frozen whole fruit** (FRWF), the **by-product** (BA), and the raw **juice** (JU). Additionally, the stability and recovery of betalains and phenolic compounds in other inedible samples, as fresh whole fruits (FRF) and peel (PE), are reported. All these inedible samples could be interesting starting materials to obtain antioxidant extracts or potential healthy nutraceuticals. Figures S2 and S3 in the Supplementary Materials showed the HPLC chromatograms of the oral, gastric, and intestinal phases during in vitro gastrointestinal digestion of the whole fruit (FWF) sample of *Opuntia stricta var. Dillenii*. Figure S2 reflects the chromatograms recorded at λ_max_ at 535 nm (Betacyanins) and 480 nm (Betaxanthins), and Figure S3 shows the chromatograms at λ_max_ at 370 nm (flavonoids) and 280 nm (Phenolic acids). The recovery (%) of these bioactive compounds in the different phases of the *in vitro* digestion was calculated as the relationship between the bioactive content in the *Opuntia Dillenii* sample and its respective content in the different phases of the digestion of the same sample, Table 2.

#### 3.4.1. Stability and Recovery of Betalains and Phenolic Compounds in Fresh Fruit Pulp and Jam (Edible Samples)

**Figure 6:**
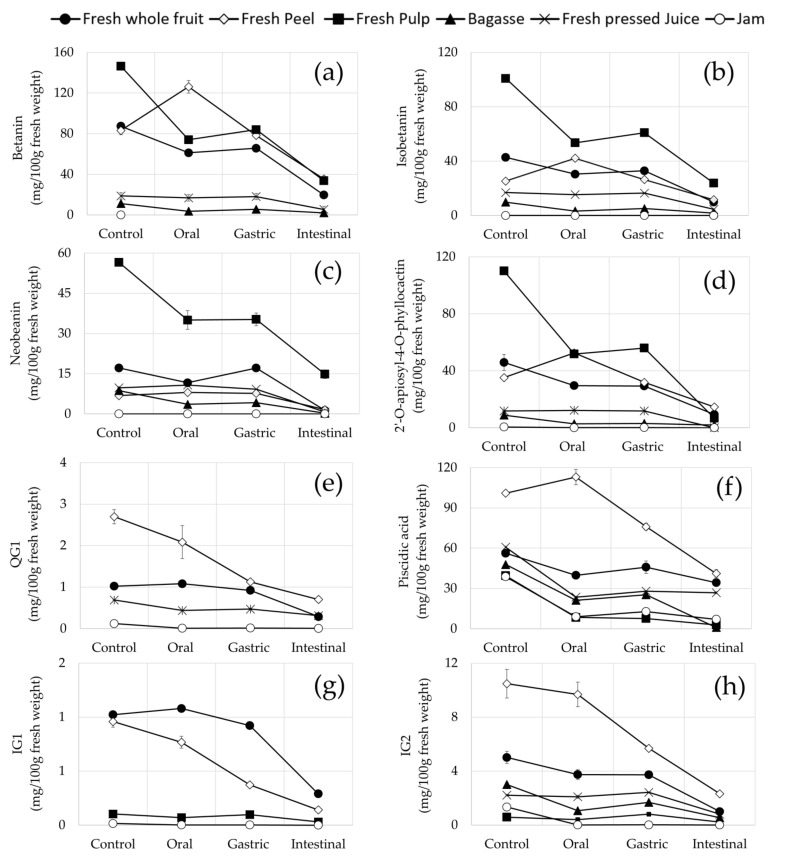
Bioactive content (mg/100 g fresh weight) in *Opuntia stricta var. Dillenii* fruit tissues (peel, pulp, and whole fruit), jam production products (intermediate juice and jam), and by-product (bagasse) during in vitro gastrointestinal static digestion. (**a**) Betanin, (**b**) isobetanin, (**c**) neobentanin, (**d**) 2′-O-apiosyl-4-O-phyllocactin, (**e**) piscidic acid, (**f**) quercetin glycoside (QG1)—quercetin hexosyl pentosyl rhamnoside, (**g**) isorhamnetin glucoxyl-rhamnosyl-rhamnoside (IG1), and (**h**) isorhamnetin glucoxyl-rhamnosyl-pentoside (IG2).

The digestive stability of betalains and phenolic compounds in *Opuntia stricta var. Dillenii* samples is shown in Figure 6, and raw data may be consulted in Supplementary Materials Table S3. Betanin from *Opuntia stricta var. Dillenii* **pulp** (PU) suffered a 42.7% loss at the end (intestinal phase) of the in *vitro* gastrointestinal digestion; meanwhile, this betalain was not present in *Opuntia* **jam** (JA) (Figure 6a). Isobetanin also showed a 40% loss during the digestion process at the intestinal phase. Other less abundant betalains such as betanidin and 2′-O-apiosyl-4-O-Phyllocactin showed losses of 53.7% and 49.1%, respectively. In contrast, Neobetanin did not suffer any significant loss during *in vitro* digestion. In *Opuntia dillenii* **jam** (JA), a loss of 84% was observed in 2′-O-apiosyl-4-O-Phyllocactin at the end of the digestion (intestinal phase) (Figure 6d). The recovery of betanin was nearly 22% in the intestinal phase in the *in vitro* digestion of fruit **pulp** (PU), and in contrast, no recovery of betanin was observed in fruit jam (JA), Table 2. This fact indicates the low stability of betanin in this *Opuntia dillenii* edible sample during *in vitro* gastrointestinal digestion, which was related to their bioaccessibility. Other *Opuntia Dillenii* abundant betalains in pulp (PU) showed different recoveries in the final phase of the digestion (intestinal phase) such as isobetanin with a recovery of 23% and 2′-O-apiosyl -4-O-Phyllocactin with a recovery of 20%, Table 2.

Regarding phenolic acids, in the *in vitro* digestion of *Opuntia stricta var. Dillenii* pulp (PU), losses of 98% in the intestinal phase and 80.6% in the gastric phase (Figure 6f) were observed, which corresponded to a piscidic content of 2.93 mg/100 g f.w. and 7.65 mg/100 g f.w., respectively, for these digestion phases (Table S3, Supplementary Materials). In the **jam** sample (JA), the content of piscidic acid during the *in vitro* digestion phases was 12.85 mg/100 g f.w. (gastric phase) and 7.02 mg/100 g f.w. (the intestinal phase), which corresponds to 67% and 82% losses, respectively.

Flavonoid compounds in *Opuntia Dillenii* **pulp** (PU) also suffered significant losses during in vitro digestion. Figure 6 showed the evolution in the content of the most abundant flavonoids in edible *Opuntia dillenii* **pulp** (PU). Relating to the recovery of phenolic compounds in edible *Opuntia Dillenii* samples, higher values were observed in pulp (PU) for isorhamnetin glucoxyl-rhamnosyl-pentoside (IG2) (20%), isorhamnetin glucoxyl-rhamnosyl-rhamnoside (IG1) (15%), and quercetin glycoside (QG1) (28%) in the intestinal phase of the *in vitro* digestion, Table 2. Similar results were reported by Gómez-Maqueo et al. [10] for the recovery of phenolic compounds of *Opuntia ficus-indica* fruits during *in vitro* gastrointestinal digestion and by Vieira Teixeira da Silva et al. [11] for pure betanin in an *in vivo* simulated gastrointestinal digestion and *ex vivo* colonic fermentation.

#### 3.4.2. Stability and Recovery of Betalains and Phenolic Compounds in Non-Edible *Opuntia stricta var. Dillenii* Samples

The study of the stability of betalains and phenolic compounds in non-edible samples such as **fresh whole fruit** (FWF), **peel** (PU), **frozen whole fruit** (FRWF), **by-product** (BA), and raw **juice** (JU) is important in order to obtain information about their potential as starting materials to obtain extracts of betalains and phenolic compounds to be used as potential healthy ingredients.

Total betalains suffered a 48.2% loss in the gastric phase and 79% in the intestinal phase during *in vitro* digestion of fresh whole fruit (FRF), Table S3 Supplementary Materials. These losses are reflected in the degradation of the most abundant individual betalain compounds at the end of the *in vitro* digestion, such as betanin (81–57%), isobetanin (77–1%), betanidin (53–92%), 2′-O-apiosyl-4-O-Phyllo- cactin (100–59%), and neobetanin (97% to 80%) (Figure 6). In the *Opuntia Dillenii* fresh whole fruit (FWF), betanin content in the oral phase and gastric phase was 61.19 mg/100 g f.w. and 65.60 mg/100 g f.w., respectively, the intestinal phase being where this betalain suffered a significant loss by degradation (78%), probably due to the intestinal environment. Betanin recoveries range from 42% to 19%, being greater in **peel** sample (PE), which was nearly 2-fold that observed for edible **pulp** (PU) at the end of the digestion (intestinal phase) (Figure 6 and Table 2). Other betalains also showed different recoveries during in vitro digestion, depending on the *Opuntia Dillenii* non-edible sample. 2′-O-apiosyl-4-O-Phyllocactin showed a null recovery in the **juice** sample (JU) (Table 2), but a higher recovery in the **peel** sample (PE), as it was observed for betanin. The lower recoveries among *Opuntia dillenii* betalains were observed for neobetanin (26% to 7%). The most important betalain′s degradation was observed at the intestinal phase, where this betalain suffered a significant degradation (up to 78%), probably due to the intestinal environmental conditions. This fact was previously reported for *Opuntia ficus-indica* edible and inedible tissue of pricky pear fruits [10] and other cactus fruits such as cactus berry fruits (*Myrtillocactus geometrizans*) [17].

Piscidic acid also suffered a considerable degradation during *in vitro* digestion of *Opuntia stricta var. Dillenii* non-edible samples (Figure 6f and Table S3). The greater loss (98%) in this phenolic acid was observed in the **by-product** sample (BA), followed by the peel sample (PE) with a stability of nearly 60% and the raw **juice** (JU) (the intermediate material for jam production, Figure 2) with a loss of 56% at the end of the *in vitro* digestion. In addition, flavonoid compounds in *Opuntia stricta var. Dillenii* non-edible samples also suffered significant losses during *in vitro* digestion. In **fresh whole fruit** (FWF), the total flavonoids showed a loss of 79%, these losses being greater in the intestinal phase of the *in vitro* digestion. The most abundant flavonoid, isorhamnetin glucoxyl-rhamnosyl-pentoside (IG2), suffered a loss of 80% in **fresh whole fruit** (FWF), a loss of 59% in **pee**l (PE), a loss of 81% in **by-product** (BA), and a loss of 63% in raw **juice** (JU) (Figure 6h and Table S3, Supplementary Materials). The two other flavonoids present in *Opuntia Dillenii* samples, isorhamnetin glucoxyl-rhamnosyl-rhamnoside (IG1) and Quercetin glycoside (QG1), also showed a significant degradation during *in vitro* digestion, being more important at the intestinal phase (Figure 6g,e). Recoveries of flavonoids in the non-edible *Opuntia Dillenii* samples ranged from 45–28% for quercetin glycoside (QG1), 30–15% for isorhamnetin glucoxyl-rhamnosyl-rhamnoside (IG1), and 41–18% for isorhamnetin glucoxyl-rhamnosyl-pentoside (IG2) in the intestinal phase. Among flavonoids, quercetin glycoside (QG1) was the most stable flavonoid compound during *in vitro* digestion in these samples, Table 2.

### 3.5. Bioaccessibility of Opuntia stricta var. Dillenii betalains and Phenolic Compounds during In Vitro Gastrointestinal Digestion

#### 3.5.1. Bioaccessibility in *Opuntia stricta var. Dillenii* Edible Samples (Pulp and Jam)

The bioaccessibility of most the abundant betalains and phenolic compounds in *Opuntia stricta var. Dillenii* edible samples after *in vitro* gastrointestinal digestion are presented in Table 3. The bioaccessibility of betanin in the **pulp** sample (PU) was nearly 23%, being zero for *Opuntia Dillenii* **jam** (JA). In the above sections, we reported that *Opuntia dillenii* jam (JA) showed a very low betalain content due to the thermal processing applied for its elaboration from raw juice. Other less abundant betalains in the fruit **pulp** (PU) (edible sample) showed different bioaccessibilities such as betanidin (47%); isobetanin (near 29%); 2′-O-apiosyl-4-O-Phyllocactin (20%); and finally, neobetanin (7.6%). *Opuntia Dillenii* **jam** (JA) also showed zero bioaccessibility in these betalains.

Bioaccessibility of piscidic acid in *Opuntia stricta var. Dillenii* **pulp** (PU) was very low, showing a value of 7.5%; meanwhile, in **jam** (JA) it was greater, at 18%, Table 3. These differences could be due to the different stability of this phenolic acid in the samples during the *in vitro* digestion. Fresh **pulp** (PU) contains enzymes that remain active during *in vitro* digestion, which could increase the action of digestive enzymes. Additionally, the differences among the pHs of these two edible *Opuntia dillenii* samples, **pulp** (PU) pH 3.55 and **jam** (JA) pH 6.2, could affect the bioaccessibility of this phenolic acid. Related to the flavonoid bioaccessibility in these edible *Opuntia Dillenii* samples, only isorhamnetin glucoxyl-rhamnosyl-pentoside (IG2) was shown to be bioaccessible in **pulp** (PU), with a value of 37.7% and a bioaccessibility of only 0.81% in *Opuntia dillenii* **jam** (JA). The other two flavonoids present in *Opuntia stricta var. Dillenii* fruits, isorhamnetin glucoxyl-rhamnosyl-rhamnoside (IG1) and quercetin glycoside (QG1), were not bioaccessible in these edible samples, Table 3.

#### 3.5.2. Bioaccessibility of Betalains and Phenolic Compounds in *Opuntia stricta var. Dillenii* Inedible Samples

Betanin bioaccessibility ranged from 42.6% in the **peel** sample (PE) to 19% in the *Opuntia Dillenii* **by-product** (BA), Table 3. This difference was also observed for the other betalain compounds such as isobetanin (45.6% to 18.5%), betanidin (25.9% to 10.3%), 2′-O-apiosyl-4-O-phyllocactin (41.3% to 21.9%), and, finally, neobetanin (23.2 to 7.2%), Table 3. These bioaccessibility values are slightly greater that those observed for betalain′s bioaccessibility in *Opuntia stricta var. Dillenii* edible samples, **pulp** (PU) and **jam** (JA). Piscidic acid bioaccessibility in *Opuntia Dillenii* inedible samples was greater in raw **juice** (JU), with a value of 44.1%, followed by the value for **peel** (PE), 40.1%, and for the **by-product** (BA), with 2%, Table 3. With respect to the bioaccessibility of flavonoids in these non-edible *Opuntia Dillenii* samples, a greater bioaccessibility value was observed for quercetin glycoside (QG1) in the **peel** sample (PE) with a value of 57.2% and for isorhamnetin glucoxyl-rhamnosyl-pentoside (IG2) in raw **juice** (JU) (the intermediate product to obtain fruit jam) with a bioaccessibility of 36.3%, Table 3.

## 4. Discussion

### 4.1. Betalain and Phenolic Compounds in Opuntia stricta var. Dillenii Fruit Samples

#### 4.1.1. Betalains in *Opuntia stricta var. Dillenii* Samples

The betalain profile of the fruits of *Opuntia stricta var. Dillenii* from Lanzarote Island (Spain) was similar to the one reported by Moussa-Ayoub et al. (2011) [19], who described that the *Opuntia dillenii* fruit peel and pulp exhibit an intensive red-purple color due to their high content of betacyanins, with betanin as the predominant compound, while betaxanthins were not found at all. Betancourt et al. (2017) [1,20] reported the presence of some betaxanthins such as tryptophan–betaxanthin and tyrosine–betaxanthin (portulacaxanthin II) in extracts of *Opuntia dillenii* (Ker-Gawl) Haw fruit from Chachagüí (Nariño, Colombia). However, these two betaxanthins were not identified in any Spanish *Opuntia stricta var. Dillenii* fruit samples analyzed in the present study. These differences in the betalain profile could be related to the different agronomical conditions of the *Opuntia* cactus growing and the intrinsic differences among cactus of different origin. Other minor betalains such as 17-decarboxy-betanin, isobetanin, betanidin, and 17-descarboxy-Neobetanin were identified in the *Opuntia dillenii* samples in the present study, these being betalain compounds previously reported by Thi Tran et al. [5] in the metabolome investigation of *Opuntia stricta var. Dillenii* fruits, recognizing them as novel fruit metabolites of betalains.

Some authors reported lower content of betanin in *Opuntia stricta* fruit pulp (80 mg/100 g f.w.) in the plentiful purple fruit color [1,19] that was observed in the Spanish *Opuntia dillenii* fruit pulp (PU) from Lanzarote (Spain), which showed a content of betanin of 146.36 mg/100 f.w. (Table S2). Spanish *Opuntia stricta var. Dillenii* also showed important amounts of 2′-O-apiosyl-4-O-Phyllocactin, such as 110.06 ± 5.50 mg/100 g f.w. in fresh **pulp** (PU)h, 45.86 ± 5.73 mg/100 g f.w. in **fresh whole fruit** (FWF), and 35.17 ± 1.76 mg/100 g f.w. in fresh **peel** (PE) (Table S2, Supplementary Materials) This betalain was not present in *Opuntia ficus-indica* fruits [4,10] but could be identified in other cactus fruits such as garambullo (*Myrtillocactus geometrizans*) [17], which contains 5876.9 µg/g dry weight. Moussa-Ayoub et al. (2016) [11] reported that *O. Dillenii* fruit juice had a high content of betacyanins and a very small amount of betaxanthins. In the present study, *any Opuntia dillenii* sample showed the presence of betaxanthin compounds.

The observed differences in the profile and content of betalain compounds among **fresh whole fruit** (FWF) and slowly **frozen whole fruit** (FRFW) (an industrial starting material for jam production) (Figure 4 and Table S2, Supplementary Materials) could be related to the different treatment applied to stabilizing the samples until analysis or processing (Figure 2). For characterization studies, the **fresh whole fruits** (FWF) were frozen by liquid nitrogen and immediately freeze-dried to obtain a lyophilized power, which was stored at −24 °C until HPLC analysis, as reported before in the Material and Methods section. Moreover, the named **frozen whole fruit** (FRFW) refers to the *Opuntia Dillenii* whole fruits, which were slowly frozen in a storage cabin at −18 °C at the Bernardo′s company (Lanzarote, Spain). These **frozen whole fruits** were stored in a frozen state until they were used as a starting material to obtain jam. This freezing process was conducted at the company, which elaborates the commercial jam in order to have a cheap and ready-to-use starting *Opuntia* fruit material for processing. In order to study this frozen whole fruit sample (FRFW), an additional freezing process using liquid nitrogen was carried out at the laboratory used in all *Opuntia* samples of the present study to block any additional degradation of bioactives before analysis, followed by a freeze-drying step and the storage of the lyophilized FRWF sample, as was performed for all other *Opuntia stricta var. Dillenii* samples studied in the present work (see the Materials and Methods section).

The betalain profile observed in the *Opuntia dillenii* **by-product** (BA) sample was similar to that of the **fresh whole fruit** (FWF), because the by-product (BA) was not submitted to thermal processing, being a residue obtained by the squeezing process to produce the raw **juice** (JU) employed to formulate the final product, the *Opuntia dillenii* **jam** (JA). A published review in 2018 reported the variability of the stability of bioactive compounds in fruit jam and jelly during processing and storage [21]. These authors described that the composition of jams and jellies has a significant role in preserving the bioactive compounds of fruits and that the optimization of the processing time and temperature is of utmost importance to retain maximum bioactives in fruit jam. However, in this review, only phenolic compounds (anthocyanins and other phenolic compounds) and ascorbic acid losses in fruit jams were reported. However, jams elaborated with betalain-rich fruits were not included in the review, because there is no previous published information about the betalain degradation in the processing of betalain-rich fruit jams.

The *Opuntia dillenii* jam (JA) did not present any amount of betanin or isobetanin due to the degradation of these compounds produced for the thermal processing, showing only low amounts of Cyclo-dopa-5-O-β-glucoside and its isomer, together with 2′-O-apiosyl-4-O-Phyllocactin and 5″-O-E-sinapoyl-2′-apyosil-Phyllocactin, Table S2. These compounds are reported also by Fernández-López et al. (2007) [22], who conducted a study about the impact of pH and temperature on the pigment pattern of cactus pear fruit extracts, showing that these processing conditions can severely affect the stability of betalains. The process to obtain *Opuntia dillenii* fruit **jam** (JA) included a thermal treatment to cook and sterilize the formulation (45% fruit juice (JU) + 1% pectin + 0.05% citric acid + 35 % sucrose) made from the fruit raw **juice** (JU) with a final sugar content of 60% in the jam. This thermal treatment was carried out at 105 °C for the cooking stage and subsequent cooling at 85 °C to make the jam filling in crystal containers. This thermal processing produced the observed degradation of betalain compounds, being also affected by the low pH of the product (≤3.5), favoring these betalain losses (Figure 4 and Table S2, Supplementary Materials).

#### 4.1.2. Phenolic Compounds in *Opuntia stricta var. Dillenii* Samples

The presence of piscidic acid and its isomers was also reported in *Opuntia ficus-indica* fruits and cladodes, and it was first quantified in *Opuntia ficus-indica* fruits by García-Cayuela et al. [4]. Recent published papers reported the presence of eucomic acid in fruits of *Opuntia ficus-indica* but their content in different fruit tissues was not reevaluated [12,18,23]. In the present study, eucomic acid and its isomers were identified in *Opuntia stricta var. Dillenii* samples, being also quantified (Table S2). In addition, almost all of the phenolic acids found in *Opuntia stricta var. Dillenii* fruit samples in the present study were previously reported for different authors in *Opuntia ficus-indica* cladodes [24], fruit juices [25], and seeds [26].

Respect to the flavonoid profile, *Opuntia stricta var. Dilleni* samples did not show the presence of isorhamnetin hexosyl-pentoside (IG6) and isorhamnetin hexoxyl-rhamnoside (IG7), which were found in other *Opuntia* fruits such as *Opuntia ficus-indica* fruits of Blanco Buenavista variety from Canary Islands (Spain) [10]. In addition, *Opuntia ficus-indica* fruits showed higher total flavonoid content than *Opuntia dillenii* fruits, as reported by several authors, reporting a total flavonoid content of 62.16 mg/100 g f.w. in fruit pulp and 11.49 mg/100 g f.w. in fruit peel of Colorada and Fresa verities of *Opuntia ficus-indica* fruits [10]. Moreover, *Opuntia stricta var. Dillenii* **pulp** (PU) and **peel** (PE) showed a content of total flavonoids of 0.79 mg/100 g f.w. and 7.39 mg/100 g f.w., respectively (Table S2, Supplementary Materials). Other authors such as Moussa-Ayoub et al. [11] reported that the *Opuntia Dillenii* cactus fruit juice exhibited desirable technological characteristics, containing a high amount of phenolic compounds, which were the major contributors to the overall antioxidant activity of the juice, and the isorhamnetin 3-O-rutinoside was only found as single flavonol in the fruit’s peel. In contrast, in the present study, three flavonoids were identified and quantified in *Opuntia dillenii* **peel** (PE) tissue, isorhamnetin glucoxyl-rhamnosyl-pentoside (IG2) (5.01 mg/100 g f.w.), quercetin glycoside (QG1) (1.31 mg/100 g f.w.), and isorhamnetin glucoxyl-rhamnosyl-rhamnoside (IG1) (0.40 mg/100 g f.w.) (Figure 5b and Table S2, (Supplementary Materials). These different compositions found in *Opuntia dillenii* peels can be related to the different agronomic conditions of the cactus fruit growing.

Another difference among fruit compositions of *Opuntia* species was the presence of ellagic acid and some of its derivatives, which were found in Spanish *Opuntia dillenii* fruits but not in Spanish *Opuntia ficus-indica* ones. As an example, in *Opuntia dillenii* **fresh whole fruit** (FWF), the total ellagic acid content was 5.80 ± 0.29 mg/100 g f.w. (Table S2). These hydrolyzable tannins were previously reported in other *Opuntia* spp. Such as *Opuntia humifusa* with a content of 1.09 (mg/g d.w.) [20].

Because of the antioxidant activity of the above-identified and quantified *Opuntia stricta var. Dillenii*, bioactive compounds have been associated with numerous health benefits. The studied *Opuntia stricta var. Dillenii* samples in this work showed an interesting profile of betalains and phenolic compounds; mainly related to their content in betanin, a betacyanin associated with *in vitro* anti-inflammatory activity and hepatic protective functions [27,28,29,30]. Prickly pears (*Opuntia ficus-indica*) and *Opuntia stricta var. Dillenii* are also sources of piscidic acid (phenolic acid) and isorhamnetin (flavonoid) mainly found as glycosides. Piscidic acid and isorhamnetin glycosides have shown anti-hypercholesterolemia effects by inhibiting cholesterol permeation *in vitro* [31], and they have been identified in *Opuntia ficus-indica* extracts with anti-inflammatory activity [14]. In the present study, as mentioned above, *Opuntia stricta var. Dillenii* samples have different amounts of flavonoids, IG1 and IG2, together with QG1. Additionally, the in vitro antioxidant, anti-inflammatory, and anti-hyperglycemic activities of isolated, purified, and semi-synthesized betalains and phenolic compounds from *Opuntia ficus-indica* prickly pear fruits have been recently reported by Gómez-Maqueo et al. [32]. Additionally, some authors reported that *Opuntia* extracts are hepatoprotective and can be used as a nutraceutical to prevent Acetaminophen (APAP)-induced acute liver failure (ALF), which is a serious health problem in developed countries [33]. Interestingly, there are data in the literature showing that *Opuntia* extracts can be considered reliable and safe since no toxicity or only low toxicity has been found in animal models [34,35]. Precisely for these reported data about betalain and phenolic compounds’ bioactivities and health-related studies, the composition of these bioactive compounds (betalains and phenolics) of *Opuntia stricta var. Dillenii* tissues and the products of their industrialization and their bioaccessibilty are interesting data to evaluate these plant materials as sources of bioactives to obtain promising healthy foods and ingredients. It is important to note that, so that these bioactive compounds can exert their beneficial effects, they (betalains and phenolic compounds) should be previously released or decompartmentalized from the cellular structures of the food material in which they are contained upon ingestion (mastication) and during the gastrointestinal digestion. For this reason, it is very important to study the bioaccessibility and stability of betalains and phenolic compounds of each *Opuntia stricta var. Dillenii* sample during the *in vitro* gastrointestinal digestion, the edible ones (fruit **pulp** (PU) and **jam** (JA)), and the inedible ones (**fresh whole fruit** (FWF), fruit **peel** (PE), **frozen whole fruit** (FRWF), **by-product** (BA), and raw **juice** (JU)) in order to get a preliminary information of the healthy potential of these products.

### 4.2. Stability and Recovery of Opuntia stricta var. Dillenii betalains and Phenolic Compounds during In Vitro Gastrointestinal Digestion

The most important loss of betalain compounds in *Opuntia dillenii* **pulp** (PU) (edible fruit tissue) during the *in vitro* gastrointestinal digestion took place at the gastric phase of the digestion (betanin loss of 42.7%) due to the low pH and the presence of the digestion enzymes (Table S3). Stability values for individual betalains from *Opunta stricta var. Dillenii* pulp (PU) (Table S2) were higher than the reported ones for several authors in other betalain-rich fruit pulps [10,17,36]. Some of these authors reported that betalains are only stable at pH from 3–7, which explains their decay in the gastric phase, where they observed losses between 21% in *Opuntia ficus-indica* Colorada pulp fruit variety [10] and <25% in red dragon fruit juice [8], which were related to the gastric-like environment. This fact was also previously reported for betalain compounds’ stability and recovery from the edible fractions of other cactus fruits such as cactus berry fruits (*Myrtillocactus geometrizans)* [17] during *in vitro* gastrointestinal digestion studies.

This observed better stability and recovery of betalains in *Opuntia dillenii* pulp (PU) was also observed for their phenolic compounds during in vitro gastrointestinal digestion (Table 2). Previous published studies on the stability of piscidic acid from *Opuntia ficus-indica* fruits of different varieties showed losses of this phenolic acid of 53–71% [10] during digestion, being slightly lower values than those obtained in the present work related to *Opuntia stricta var. Dillenii* (59–97%). These differences could be due to the presence of other constituents in the *Opuntia dillenii* fruit **pulp** (PU) that contributed to the phenolic compounds’ stability such as the presence of high total fiber content, consisting of high amounts of pectin and mucilage [37]. Flavonoid compounds in *Opuntia Dillenii* **pulp** (PU) also suffered significant losses during *in vitro* digestion. In this sample, isorhamnetin glucoxyl–rhamnosyl–pentoside (IG2) suffered a loss of 64.4% in the intestinal phase and 99% in *Opuntia dillenii* jam (JA). This fact could be due to the very low content of flavonoids in the *Opuntia dillenii* **jam** (JA).

In the studied non-edible samples of *Opuntia dillenii* fruits (**fresh whole fruit** (FWF), **peel** (PU), **frozen whole fruit** (FRWF), **by-product** (BA), and raw **juice** (JU), significant losses in the content of betalain compounds during *in vitro* gastrointestinal digestion were also observed as the same as what happened in the edible samples, reflecting the degradation of the most abundant individual betalain compounds due to the environment in the digestion phase. The observed losses of betalain compounds (betanin (81–57%), isobetanin (77–1%), betanidin (53–92%), 2′-O-apiosyl-4-O-Phyllo-cactin (100–59%), and neobetanin (97% to 80%)) (Figure 6) at the end of the *in vitro* digestion were higher that those reported by different authors for these compounds in other betalain-rich fruits [10,17,37]. Tesorière et al. [8] also reported a similar loss of betacyanins, either purified or food-derived from cactus pear fruit (*Opuntia ficus indica* L. Mill. cv. Gialla and Rossa) and red beet (*Beta vulgaris* L. ssp. *vulgaris*), indicating that the release of betacyanins from the matrix was incomplete at the intestinal phase due to additional factors relevant to the food matrix and style of processing of the fruit that affect the betacyanin′s stability and bioaccessibility.

The greater loss (98%) of piscidic acid was observed in the **by-product** sample (BA), which could be attributed to the physical cell rupture of plant material produced during processing (squeezing process) to obtain the raw juice for jam production. During the squeezing process, the liberation of tissue oxidative enzymes could take place and could increase the action of the digestive enzymes on the phenolic compounds. Moreover, in other processed *Opuntia dillenii* inedible samples, such as the raw **juice** (JU) (the intermediate material for jam production, Figure 2), the loss of piscidic acid was only 56% at the intestinal phase, and in the **peel** sample (PE), the stability of piscidic acid was nearly 60% at the end of the *in vitro* digestion. This better stability of piscidic acid in these samples could be due to their polysaccharide composition, which could protect the phenolic acid from the digestive milieu. This fact was previously reported by Gómez-Maqueo et al. [10] in the study of the stability of phenolic compounds of *Opuntia ficus-indica* fruit peels of different fruit varieties, indicating losses of piscidic acid of 45%. These authors suggested that *Opuntia ficus-indica* peels could be proposed as potential by-products to obtain sustainable healthy ingredients. Results from the present study about *Opuntia stricta var. Dillenii* agree with this conclusion, the fruit **peel** (PE) being the most interesting material to use for extraction of antioxidant bioactives due to its composition in bioactive compounds and the observed higher stability of them during *in vitro* digestion. Additionally, in this reported study, the recoveries of flavonoids in *Opuntia ficus-indica* fruit **peels** were 46–64% for isorhamnetin glucoxyl-rhamnosyl-rhamnoside (IG1) and 52–70% for isorhamnetin glucoxyl-rhamnosyl-pentoside (IG2), respectively, during *in vitro* digestion, depending on the fruit variety. These values are higher than those obtained in the present study of *Opuntia stricta var. Dillenii* inedible samples such as peel (PE) (IG1 30.6% and IG2 41.4%), possibly due to the different composition in other constituents of the fruit tissue as polysaccharides could influence the degradation processes of flavonoids during *in vitro* gastrointestinal digestion, as was mentioned before for betacyanin degradation. The stability of phenolic compounds during digestion partially depends on their glycosylation pattern; for instance, isorhamnetin glycosides from *Opuntia ficus-indica* cladodes are more bioaccessible than their respective aglycones [14].

### 4.3. Bioaccessibility of Opuntia stricta var. Dillenii betalains and Phenolic Compounds during In Vitro Gastrointestinal Digestion

In *Opuntia stricta var. Dillenii* edible **pulp** (PU), no metabolites or degraded products could be detected by the HPLC method employed in the present work during the different phases of the *in vitro* gastrointestinal digestion. This fact agrees with the literature reporting that betanin compounds are not metabolized during digestion or in the liver [38,39]. A deeper metabolomic study must be carried out in order to amply this information about the bioavailability of *Opuntia stricta var. Dillenii* betalains. Gómez-Maqueo et al. [10] reported that betanin showed different bioaccessibility values in *Opuntia ficus-indica* pulps, depending on the fruit variety. In this published study, betanin bioaccessibility in Colorado and Fresa fruit prickly pear fruit pulps were 46% and 45%, respectively; however, betanin bioaccessibility in Blanco Fasnia and Blanco Buenavista fruit pulps were only 17% and 9.6%, respectively. In the present study about *Opuntia stricta var. Dillenii*, betanin bioaccessibility in **pulp** (PU) was of 23%; this value was similar to the reported bioaccessibility values for betanin from *Opuntia ficus-indica* pulps of Blanco fruit variety. An important fact conclusion from these data is that the commercial production of *Opuntia Dillenii* jam must be modified in order to try to avoid the thermal degradation of betalains and phenolic compounds, them being more bioaccessible and facilitating that these bioactive compounds will reach the intestinal phase and will be absorbed in the epithelial cells of the intestine [40], and later they will exert its bioactivity [38]. Other processing technologies for sterilization must be proposed to preserve antioxidant bioactives in *Opuntia* jam production.

The bioaccessibility data for phenolic compounds observed for *Opuntia stricta var. Dillenii* non-edible samples (Table 3) were lower than those observed for these same phenolic compounds from fruit peels of different Spanish *Opuntia ficus-indica* varieties, as reported by Gómez-Maqueo et al. [10], and from Italian pricky fruit varieties, as reported by Tesorière et al. [8]. These authors reported that the prickly pear peels are rich in phenolic compounds such as piscidic acid, 4-Hydroxy-benzoic acid derivative, and also in different isorhamnetin glycosides that showed high digestive stability and bioaccessibility. In the present work, among the studied *Opuntia stricta var. Dilleni* non-edible samples, the fruit **peel** (PE) was the most interesting material to obtain antioxidant extracts due to its content being rich in bioaccessible betalains (45.6–21.7%) and phenolic compounds (53.7–30.6%), followed by the raw **juice** (JU), with bioaccesibility values for phenolic compounds (45.3–29.4%) similar to those previousky reported for peel tissue of Colorado and Fresa varieties of *Opuntia ficus-indica* [10]. However, *Opuntia dillenii* **by-product** (BA) would not be recommended as a starting cactus fruit material to extract bioactives due to the observed low stability of them during *in vitro* digestion and their observed low bioaccessibility.

## 5. Conclusions

A total of 60 betalain and phenolic compounds were identified and quantified in *Opuntia stricta var. Dillenii* samples, including 25 phenolic acids (including isomers and derivatives), 12 flavonoids (including glycosides), 3 ellagic acids (including glycosides and derivative), and 20 betanins (including degradation compounds). The greatest bioaccessibility values were observed in fruit peel (PE) with values for betanin (42.5%) and quercetin glycoside (QG1) (53.7%). Moreover, in *Opuntia Dillenii* pulp (the edible fraction of fresh fruit), the betanin bioaccessibility was only 22.9%, and flavonoids’ bioaccessibility ranged from 53.7% to 30.6%. betanin bioaccessibility in commercial *Opuntia dillenii* jam was zero, and very low values were observed for some phenolic compounds as piscidic acid (18.1%), quercetin glycoside (QG1) (7.1%), and isorhamnetin glucoxyl-rhamnosyl-pentoside (IG2) (0.8%). The results of the present study indicate that an alternative process must be proposed to obtain *Opuntia stricta var. Dillenii* jams to avoid the betalain and phenolic compund degradation during processing. *Opuntia stricta var. Dillenii* fruit peel (PE) and fresh whole fruit (FWF) are the most interesting materials to obtain antioxidant extracts rich in betalains and phenolic compounds with great bioaccessibility values. In contrast, the by-product (BA) from jam processing was not so interesting to use as a starting material to obtain bioactive extracts due to its lower content of betalains (44.14 mg/100 g f.w.) and phenolic compounds (87.17 mg/100 g f.w.) and its low bioaccessibility.

## Figures and Tables

**Table 1:** HPLC retention times (Rt), U*V/V*is spectra, and MS/MS spectral data of betalains, phenolic compounds, and organic acids in *Opuntia stricta, var. Dillenii* fruit tissues (peel, pulp, and whole fruit), jam production products (intermediate juice and jam), and by-product (bagasse).

Peak *	t_R_ (min)	Compounds	UV λmax (nm)	[M-H]^+^	[M-H]^−^	MS/MS (*m/z)*	Samples ^1^
1	3.17	Pyruvic acid	230		87	59.01	FWF, JU, BA
2	3.31	Gallic acid derivative	232, 271		331	271, 211, 169, 151, 125, 113, 89, 71, 59	FWF, JU, BA, JA
3	3.47	Ascorbic acid	285		175	115, 89	FWF, PE, PU, FRFW, JU, BA, JA
4	3.59	Piscidic acid isomer I	272		255	193, 165, 135, 119 107	FWF, PE, PU, JU, BA, JA
5	3.75	Piscidic acid isomer II	278		255	193, 165, 135, 119 107	FWF, PE, PU, JU, BA; JA
6	4.13	Citric acid	233		191	111, 87, 67	FWF, JU
7	4.31	Ferulic acid derivative I	233		489	295,235,193,175,149	FWF,
8	4.49	Unknown	241		--	--	FWF,
9	4.70	Ferulic acid derivative II	231		489	235,193,175,149	FWF,
10	5.18	Protocatechuic acid derivative	230 (sh), 284	297		286, 153, 86	FWF, PU
11	5.46	Quinic acid	230(sh), 272		191	111, 85, 67	FWF, JU,
12	6.24	Gallic acid	274		169	125, 107, 97, 79, 69, 51, 41	FWF, PE, JU, BA
13	7.17	p-hydroxybenzoic acid	262	301		139, 121	FWF,
14	8.24	Eucomic acid derivative I	280		525	295, 235, 239, 195, 179	FWF, PU,
15	8.83	Piscidic acid	272		255	193, 165, 135, 119 107	FWF, FRWF, PE, PU, JU, BA, JA
16	9.42	15,17-bidecarboxy-betanin	480	463		301	FRWF, JA
17	9.73	Eucomic acid isomer I	280		239	195,179,149,133	FWF, JA
18	10.76	Betanin	535	551		390, 389	FWF, FRWF, PE, PU, JU, BA
19	11.67	Piscidic acid derivative I	275		487	255, 193, 165, 135, 107	FRWF, PE, JA
20	11.73	17-decarboxy-betanin	504	507		390, 389	FWF, FRWF, PE, PU, JU, BA
21	12.97	Cyclo-dopa-5-O-β-glucoside	230, 275	358		196	JA
22	14.42	Cyclo-dopa-5-O-α-glucoside (isomer)	282	358		196	JA
23	15.23	Isobetanin	535	551		390, 389	FWF, FRWF, PE, PU, JU, BA
24	15.48	17-decarboxy-isobetanin	504		505	461	JU, BA
25	19.85	Eucomic acid	278		239	195, 179, 149, 133	FWF, PE, PU
26	24.91	Betanidin	538	389		345, 150	FWF, PU, JU, BA
27	25.42	6′-O-sinapoyl-O-gompherin	539		755	225	FWF, PE, PU, JU, BA
28	26.70	Eucomic acid isomer II	280		239	195, 179, 149, 133	FWF,
29	27.90	2′-O-apiosyl-4-O-phyllocactin	537		767	551	FWF, PE, PU, JU, BA, JA
30	28.97	Unknown	331				FWF, JU, BA
31	29.08	5″-O-*E*-sinapoyl-2′-apyosil-phyllocactin	248,330,540		975	---	FWF, PE, PU, JU, BA, JA
32	30.27	17-Descarboxy-neobetanin	451	505		343	FWF,
33	31.34	Neobetanin	467		549	387	FWF, FRWF, PE, PU, JU, BA
34	31.84	Neobetanin isomer I	465		549	387	PU
35	33.16	4-Hydroxybenzoic acid 4-O-glucoside ^c^	270		299	137, 119, 93	FWF, JA,
36	33.72	Ferulic acid	297, 328	193.1		177, 161, 133	FWF, PE,
37	34.20	4-Hydroxybenzoic acid 4-O-glucoside isomer ^c^	267		299	137, 119, 93	FWF, PE,
38	35.14	Quercetin-3-O-rhamnosyl-rutinoside (QG3)	358	757		611, 303	FWF,
39	35.49	Neobetanin isomer II	449		549	387	FWF, JU, BA
40	35.82	Ellagic acid derivative I	256, 296(sh) 352		1085	479, 300, 273	FWF,
41	36.15	Ferulic acid derivative ^c^	245, 327		355	239,193,175	FWF, FWF,
42	36.40	Neobetanin isomer III	444		549	387	FWF, JU, BA
43	36.71	p-coumaric acid	280, 312		165	166, 187	FWF,
44	36.78	Protocatechuic acid derivative	356	498		137, 111, 109, 97	FWF,
45	37.00	p-coumaric acid derivative ^c^	237, 318		191	145, 119, 45, 27	FWF,
46	37.39	15R/15S-Betanidin	530	389		371, 342, 297, 194, 150, 132	FWF, FRWF, PE, JU, BA
47	37.95	Ellagic acid	366, 255	303	301	285, 283, 257, 229, 184, 134	FWF, PE; JU, BA
48	38.32	Ellagic acid rhamnoside	258, 352		447	352, 262, 160, 146	FWF
49	38.54	Myricetin	255, 372	319		153, 113	FWF
50	38.65	Quercetin hexosyl pentosyl rhamnoside (QG1)	255,358	426		303, 191, 120	FWF, FRWF, PE, JU, BA, JA
51	38.72	Myricitrin (myricetin 3-rhamnoside)	255, 374	465		319, 147	FWF
52	39.56	Quercetin glycoside (QG2)—Quercetin hexose pentoside	255, 353	653		303, 177	FWF, FRWF
53	40.00	Isorhamnetin glucoxyl-rhamnosyl-rhamnoside (IG1)	256, 356	771		625; 317, 85	FWF, FRWF, PE, PU, JU, JA
54	42.57	Isorhamnetin glucoxyl-rhamnosyl-pentoside (IG2)	254, 356	757		317, 167, 86	FWF, FRWF, PE, PU, JU, BA, JA
55	42.85	Isorhamnetin hexosyl-hexosyl-pentoside (IG3)	353	757		317	FWF
56	43.08	Rutin (quercetin-3-rutinoside) ^a^	352, 299(sh)	611		303, 229, 137	FWF
57	43.32	Isorhamnetin glucosyl-pentoside (IG4) ^a^	352, 293(sh)	611		479, 317, 177	FWF
58	45.54	Kaempferol-glucosyl-rhamnoside (KG1)	356	595	597	287	FW,
59	45.82	Isorhamnetin glucosyl-rhamnoside (IG5)	353	625		317, 85	FW,
60	46.19	Isorhamentin glucoside ^b^	330, 299(sh)	814		641, 317, 169	FW,

* Peak numbers are according to Figure 2; ^a^ confirmed and quantified with semi-synthetized, purified, or commercial standard. ^b^ quantified using a related compound with similar mass and chemical characteristics. ^c^ tentatively identified; ^1^ *Opuntia stricta va. Dillenii* samples: **FWF**, fresh whole fruit; **FRWF**, frozen whole fruit; **PU**, fresh fruit pulp; **PE**, fresh fruit peel; **JU**, fresh pressed juice (intermediate product of jam processing). **BA**, by-product of jam processing, and **JA**, fruit jam (final product of jam processing). See Figure 2.

**Table 2:** Recovery (%) of the most abundant betalain and phenolic compounds in *Opuntia stricta var. Dillenii* fruit tissues (peel, pulp, and whole fruit). Jam production products (intermediate juice and jam) and by-products (bagasse) during in vitro gastrointestinal static digestion.

		Recovery (%)					
Compound	In Vitro phase	Fresh Whole Fruit (FWF)	Fresh Peel (PE)	Fresh Pulp (PU)	Bagasse By-Product (BA)	Fresh Pressed Juice (JU)	Jam (JA)
**BETALAINS**							
Betanin	Oral	80.10 ± 2.79 ^Bc^	101.71 ± 7.59 ^Ce^	60.58 ± 1.90 ^Bb^	52.25 ± 1.06 ^Ba^	99.43 ± 1.52 ^Bd^	n.d.
	Gastric	75.16 ± 0.88 ^Bc^	94.00 ± 1.69 ^Bd^	57.31 ± 0.27 ^Cb^	49.87 ± 1.60 ^Ca^	96.34 ± 2.03 ^Bd^	n.d.
	Intestinal	22.42 ± 1.29 ^Aab^	42.58 ± 3.35 ^Ac^	22.95 ± 0.01 ^Aab^	19.03 ± 1.14 ^Aa^	28.53 ± 2.19 ^Ab^	n.d.
Isobetanin	Oral	71.07 ± 3.63 ^Bc^	166.33± 8.32 ^Ce^	53.03 ± 2.01 ^Bb^	33.54 ± 1.27 ^Ba^	90.98 ± 1.61 ^Bd^	n.d.
	Gastric	76.78 ± 0.25 ^Bc^	104.11 ± 3.27 ^Be^	60.38 ± 0.18 ^ab^	52.11 ± 1.99 ^Ca^	97.22 ± 2.11 ^Bd^	n.d.
	Intestinal	22.85 ± 1.72 ^Ab^	45.67 ± 1.66 ^Ac^	23.60 ± 0.06 ^Ab^	18.52 ± 1.20 ^Aa^	26.44 ± 2.42 ^Ab^	n.d.
Betanidin	Oral	155.11 ± 0.02 ^Be^	78.52 ± 3.93 ^Cb^	84.72 ± 2.75 ^Bc^	30.90 ± 0.18 ^Ba^	109.82 ± 2.50 ^Bd^	n.d.
	Gastric	157.49 ± 10.63 ^Be^	46.49 ± 2.32 ^Ba^	91.85 ± 1.55 ^Cb^	45.78 ± 2.22 ^Ca^	112.93 ± 1.42 ^Bc^	n.d.
	Intestinal	47.06 ± 0.77 ^Ae^	21.74 ± 1.09 ^Ab^	36.37 ± 0.35 ^Ad^	10.38 ± 0.28 ^Aa^	25.95 ± 3.14 ^Ac^	n.d.
2′-O-apiosyl-4-O-phyllocactin	Oral	65.09 ± 4.59 ^Bd^	149.40 ± 7.49 ^Cf^	47.00 ± 1.61 ^Bc^	31.01 ± 0.34 ^Bb^	103.51 ± 2.11 ^Ae^	10.38 ± 1.35 ^Ba^
	Gastric	65.09± 4.05 ^Bc^	90.58 ± 0.48 ^Bd^	50.85 ± 0.01 ^Cc^	34.61 ± 1.73 ^Cb^	100.74 ± 2.85 ^Ad^	16.12 ± 0.88 ^Ca^
	Intestinal	20.42 ± 4.04 ^Ab^	41.32 ± 0.78 ^Ac^	5.97 ± 0.30 ^Aa^	21.94 ± 0.71 ^Ab^	0	0
Neobetanin	Oral	67.96 ± 9.33 ^Bb^	116.94 ± 5.85 ^Cc^	61.92 ± 6.48 ^Bb^	41.14 ± 1.63 ^Ba^	110.47 ± 2.57 ^Cc^	n.d.
	Gastric	60.08 ± 9.70 ^Cbc^	112.27 ± 5.61 ^Bc^	62.41 ± 4.45 ^Ba^	48.74 ± 3.13 ^Ca^	94.78 ± 3.16 ^Bb^	n.d.
	Intestinal	7.63 ± 2.96 ^Ab^	23.26 ± 1.16 ^Ac^	26.22 ± 2.90 ^Ac^	3.08 ± 0.15 ^Aab^	7.20 ± 0.52 ^Ab^	n.d.
**PHENOLICS ACIDS**							
Piscidic acid	Oral	70.82 ± 2.74 ^Bd^	111.92 ± 5.60 ^Ce^	21.39 ± 0.96 ^Ba^	44.39 ± 0.87 ^Bc^	33.80 ± 1.19 ^Ab^	23.16 ± 2.02 ^Aa^
	Gastric	81.49 ± 7.81A ^Bc^	75.26 ± 2.39 ^Bc^	19.34 ± 0.74 ^Ba^	53.09 ± 1.72 ^Cb^	46.11 ± 0.19 ^Ab^	33.20 ± 0.79 ^Bb^
	Intestinal	61.35 ± 3.07 ^Ae^	40.71 ± 2.78 ^Ad^	7.43 ± 0.35 ^Ab^	2.03 ± 0.08 ^Aa^	44.17 ± 1.78 ^Ad^	18.13 ± 0.97 ^Ac^
**FLAVONOIDS**							
Quercetin glycoside(QG1)—Quercetin hexosyl pentosyl rhamnoside	Oral	105.65 ± 0.07 ^Cd^	137.98 ± 6.90 ^Ce^	n.d.	0	63.53 ± 0.44 ^Bc^	9.86 ± 0.92 ^Bb^
	Gastric	90.21 ± 0.84 ^Bd^	86.35 ± 1.72 ^Bd^	n.d.	0	68.36 ± 2.54 ^Bc^	14.76 ± 0.80 ^Cb^
	Intestinal	28.33 ± 2.18 ^Ab^	53.72 ± 5.19 ^Ad^	n.d.	0	45.38 ± 1.78 ^Ac^	7.15 ± 0.36^Aa^
Isorhamnetin glucoxyl-rhamnosyl-rhamnoside (IG1)	Oral	81.74 ± 2.07 ^Cc^	158.05 ± 7.54 ^Cd^	0	n.d.	68.15 ± 8.04 ^Bb^	11.39 ± 2.81 ^Ba^
	Gastric	64.82 ± 2.28 ^Bc^	80.77 ± 1.00 ^Bd^	0	n.d.	94.36 ± 0.91 ^Ce^	16.36 ± 3.49 ^Bb^
	Intestinal	15.27 ± 0.88 ^Ab^	30.62 ± 0.98 ^Ac^	0	n.d.	29.43 ± 3.51 ^Ac^	0.00 ± 0.00 ^Aa^
Isorhamnetin glucoxyl-rhamnosyl-pentoside (IG2)	Oral	75.43 ± 14.17 ^Bc^	148.28 ± 7.41 ^Cd^	68.56 ± 12.80 ^Ac^	35.45 ± 1.44 ^Bb^	94.44 ± 2.76 ^Bc^	0.94 ± 0.20 ^Aa^
	Gastric	74.74 ± 4.14 ^Bc^	101.38 ± 0.97 ^Bd^	136.88 ± 4.33 ^Be^	56.20 ± 2.75 ^Cb^	109.25 ± 0.90 ^Cd^	1.33 ± 0.20 ^Aa^
	Intestinal	20.02 ± 2.95 ^Ab^	41.40 ± 1.09 ^Ac^	37.77 ± 1.85 ^Ac^	18.49 ± 1.85 ^Ab^	36.33 ± 1.34 ^Ac^	0.81 ± 0.24 ^Aa^

Results were expressed as mean ± standard deviation (*n* = 4). This came from obtaining at least two independent extracts (*n* = 2) and performing the HPLC determinations of each two times (*n* = 2). Superscript capital letters indicate statistically significant differences (*p* ≤ 0.05) between digestion phases. Superscript small letters indicate statistically significant differences (*p* ≤ 0.05) between tissues, products, and by-products.

**Table 3:** Bioaccessibility of the most abundant betalain and phenolic compounds in *Opuntia stricta var. Dillenii* fruit tissues (peel, pulp, and whole fruit), jam production products (intermediate juice and jam), and by-product (bagasse) during in vitro gastrointestinal static digestion.

		Bioaccessibility (%)					
Compound		Fresh Whole Fruit (FWF)	Fresh Peel (PE)	Fresh Pulp (PU)	Bagasse By-Product (BA)	Fresh Pressed Juice (JU)	Jam (JA)
**BETALAINS**							
Betanin		22.42 ± 1.29 ^ab^	42.58 ± 3.35 ^c^	22.95 ± 0.01 ^ab^	19.03 ± 1.14 ^a^	28.53 ± 2.29 ^b^	0
Isobetanin		22.85 ± 1.72 ^ab^	45.67 ± 1.66 ^c^	23.60 ± 0.06 ^ab^	18.52 ± 1.20 ^a^	26.44 ± 2.42 ^b^	0
Betanidin		47.06 ± 0.77 ^d^	21.74 ± 1.09 ^b^	36.37 ± 0.35 ^c^	10.38 ± 0.28 ^a^	25.95 ± 3.14 ^b^	0
2′-O-apiosyl-4-O-phyllocactin		20.42 ± 4.04 ^c^	41.32 ± 0.78 ^d^	5.97 ± 0.30 ^b^	21.94 ± 0.71 ^c^	0	0
Neobetanin		7.63 ± 2.96 ^a^	23.26 ± 1.16 ^b^	26.22 ± 2.90 ^b^	3.08 ± 0.15 ^a^	7.20 ± 0.52 ^a^	0
**PHENOLICS ACIDS**							
Piscidic acid		61.35 ± 3.07 ^e^	40.71 ± 2.78 ^d^	7.43 ± 0.35 ^b^	2.03 ± 0.08 ^a^	44.17 ± 1.78 ^d^	18.13 ± 0.97 ^c^
**FLAVONOIDS**							
Quercetin glycoside(QG1)—Quercetin hexosyl pentosyl rhamnoside		28.33 ± 2.18 ^b^	53.72 ± 5.19 ^d^	0	0	45.38 ± 1.78 ^c^	7.15 ± 0.36 ^a^
Isorhamnetin glucoxyl-rhamnosyl-rhamnoside(IG1)		15.27 ± 0.88 ^b^	30.62 ± 0.98 ^c^	0	0	29.43 ± 3.51 ^c^	0
Isorhamnetin glucoxyl-rhamnosyl-pentoside(IG2)		20.02 ± 2.95 ^b^	41.40 ± 1.09 ^c^	37.77 ± 1.85 ^c^	18.49 ± 1.85 ^b^	36.33 ± 1.34 ^c^	0.81 ± 0.24 ^a^

Results were expressed as mean ± standard deviation (*n* = 4). This came from obtaining at least two independent extracts (*n* = 2) and performing the determinations of each two times (*n* = 2). Superscript small letters indicate statistically significant differences (*p* ≤ 0.05) between tissues, products, and by-products.

## Supplementary Materials

The following are available online at https://www.mdpi.com/article/10.3390/foods10071593/s1, Figure S1: Betalains, phenolic acids and flavonoids distribution (%) from *Opuntia stricta var. Dillenii* fruit tissues (peel, pulp and whole fruit). jam production products (intermediate juice and jam) and by-product (bagasse), Figure S2: HPLC-DAD chromatograms of betalains compounds recorded at 535 and 480 nm, betacyanins and betaxanthins, respectively, obtained from *Opuntia stricta var. Dillenii* whole fruit after in vitro static gastrointestinal digestion. ^1^ Numbers correspond to the identified compounds indicated in Table 1, Figure S3: HPLC-DAD chromatograms of phenolic compounds recorded at 280 and 370 nm, phenolic acids and flavonoids, respectively, obtained from *Opuntia stricta var. Dillenii* whole fruit after in vitro static gastrointestinal digestion. ^1^ Numbers correspond to the identified compounds indicated in Table 1. Table S1: Physico-chemical characteristics of *Opuntia stricta var. Dillenii* fruits from Canary Islands, Table S2: Individual betalain and phenolic compound content (mg/100 g fresh weight) in *Opuntia stricta var. Dillenii* fruit tissues (peel, pulp and whole fruit), jam production products (intermediate juice and jam) and by-product (bagasse), Table S3: Stability of the most abundant betalain and phenolic compounds in *Opuntia stricta var. Dillenii* fruit tissues (peel, pulp and whole fruit), jam production products (intermediate juice and jam) and by-product (bagasse) during in vitro gastrointestinal static digestion.
