# Supplementary material for: Characterization, Stability, and Bioaccessibility of Betalain and Phenolic Compounds from Opuntia stricta var. Dillenii Fruits and Products of Their Industrialization
Source: Foods. 2021 Jul 9;10(7):1593. doi: 10.3390/foods10071593 (PMC8307708; doi:10.3390/foods10071593)
Supplement: Supplementary file 1 [file foods-10-01593-s001.zip › foods-1290788-supplementary.pdf]

Table S1. Physico-chemical characteristics of *Opuntia stricta var. Dillenii* fruits from Canary Islands.

**Physicochemical characteristics**	***Opuntia dillenii whole fruit***
Apical caliber (cm)	6.10 ± 0.60
Equatorial caliber (cm)	3,96 ± 0,14
Fruit Weight (g)	54,35 ± 6,82
% Peel	14.26 ± 0,79
% Pulp	52,94 ± 1,13
% Seeds	32,80 ± 0,46
pH	3,55 ± 0,08
Soluble solids (ºBrix)	10,80 ± 0,30
Titratable acidity (%)	1,58 ± 0,10
Moisture (%)	81,83 ± 4,09
Color pulp (CIELAB)	
L*	30,92 ± 1,39
a*	4,477 ± 3,26
b*	-7,46 ± 0,51
Color peel (CIELAB)	
L*	29,03 ± 0,67
a*	4,07 ± 0,69
b*	-8,09 ± 0,91

Result were expressed as mean ± standard deviation (n=2).

Table S2. Individual betalain and phenolic compound content (mg/100g fresh weight) in *Opuntia stricta var. Dillenii* fruit tissues (peel, pulp and whole fruit), jam production products (intermediate juice and jam) and by-product (bagasse)

	Tissues						
**Compound**	**Fresh whole fruit (FWF)**	**Fresh Peel (PE)**	**Fresh Pulp (PU)**	**Frozen whole fruit (FRWF)**	**Bagasse By-product (BA)**	**Fresh pressed Juice (JU)**	**Jam (JA)**
**BETALAINS**							
15,17-bidecarboxy-betanin	-	-	-	5.05 ± 0.27^{a}	.	.	.
Betanin	87.28 ± 0.96^{d}	83.12 ± 4.16^{c}	146.36 ± 2.08^{e}	20.82 ± 0.70^{b}	11.21 ± 0.18^{a}	18.77 ± 0.02^{b}	-
17-decarboxy-betanin	1.18 ± 0.06^{a}	n.d.	n.d.	n.d.	2.44 ± 0.12^{b}	3.26 ± 0.16^{c}	-
Cyclo-dopa-5-O-β-glucoside	-	-	-	-	-	-	0.57 ± 0.01^{a}
Cyclo-dopa-5-O-α-glucoside (isomer)	-	-	-	-	-	-	0.06± 0.00^{a}
Isobetanin	42.78 ± 0.04^{e}	25.31 ± 1.27^{c}	100.77 ± 1.48^{f}	31.42 ± 0.09^{d}	9.78 ± 0.16^{a}	16.88 ± 0.01^{b}	-
17-decarboxy-isobetanin	-	-	-	-	2.05 ± 0.05^{a}	2.71 ± 0.02^{b}	
Betanidin	1.15 ± 0.11^{a}	4.32 ± 0.22^{a}	1.70 ± 0.09^{a}	-	0.24 ± 0.01^{a}	0.45 ± 0.01^{a}	-
6´-O-sinapoyl-O-gompherin	1.59 ± 0.02^{d}	2.01 ± 0.16^{e}	2.81 ± 0.14^{e}	0.89 ± 0.16^{c}	0.21 ± 0.00^{a}	0.45 ± 0.02^{b}	-
2´-O-apiosyl-4-O-phyllocactin	45.86 ± 5.73^{d}	35.17 ± 1.76^{d}	110.06 ± 5.50^{e}	15.45 ± 0.04^{c}	8.83 ± 0.08^{b}	11.75 ± 0.40^{b}	0.51 ± 0.03^{a}
5´´-O-*E*-sinapoyl-2´-apyosil-phyllocactin	16.09 ± 0.70^{d}	14.69 ± 0.73^{c}	26.50 ± 1.32^{e}	4.21 ± 0.26^{b}	n.d.	2.30 ± 0.07^{a}	0.53 ± 0.03^{a}
17-Descarboxy-neobetanin	0.39 ± 0.03a	-	-	-	-	-	-
Neobetanin	17.17 ± 0.70^{e}	6.85 ± 0.34^{b}	56.57 ± 0.29^{f}	10.78 ± 0.01^{a}	8.71 ± 0.19^{c}	9.74 ± 0.27^{d}	-
Neobetanin isomer I	-	-	n.d.	-	-	-	-
Neobetanin isomer III	1.16 ± 0.06^{c}	-	-	0.35 ± 0.02^{b}	0.27 ± 0.04^{a}	0.44 ± 0.00^{c}	-
Neobetanin isomer III	0.96 ± 0.05^{c}	-	-	0.71 ± 0.04^{c}	0.41 ± 0.05^{a}	0.69 ± 0.06^{b}	-
15R/15S-Betanidin	0.27 ± 0.01^{b}	0.36 ± 0.02^{c}	-	0.09 ± 0.00^{a}	n.d.	n.d.	-
**Total betalains**	212.91 ± 10.65^{b}	171.83 ± 8.59^{b}	444.77± 22.84^{c}	89.77 ± 4.49^{a}	44.14 ± 2.21^{a}	67.45 ± 3.37^{a}	1.67 ± 0.08^{a}
**PHENOLICS ACIDS**							
Gallic acid derivative	0.60 ± 0.10^{c}	-	-	0.38 ± 0.01^{b}	0.48 ± 0.00^{c}	0.47 ± 0.02^{c}	0.02 ± 0.00^{a}

Piscidic acid	56.21 ± 0.30^{c}	101.00 ± 5.04^{e}	39.52 ± 0.65^{a}	59.67 ± 0.37^{c}	47.85 ± 0.27^{b}	60.74 ± 0.70^{d}	38.71 ± 2.13^{a}
Piscidic acid isomer I	79.65 ± 3.66^{b}	65.12 ± 3.26^{a}	n.d.	-	6.38 ± 0.41^{a}	93.62 ± 1.58^{c}	n.d.
Piscidic acid isomer II	72.54 ± 1.54^{d}	62.96 ± 3.15^{d}	79.43 ± 2.01^{e}	12.23 ± 0.19^{c}	13.38 ± 0.37^{b}	14.92 ± 0.12^{b}	10.43 ± 0.66^{a}
Piscidic acid derivative I	-	38.90 ± 1.92^{b}	-	4.00± 0.21^{a}	-	-	n.d.
Ferulic acid	0.33 ± 0.01^{a}	0.23 ± 0.01^{b}	-	-	-	-	-
Ferulic acid derivative I	0.52 ± 0.03^{a}	-	-	-	-	-	-
Ferulic acid derivative II	0.56 ± 0.07^{a}	-	-	-	-	-	-
Ferulic acid derivative III	0.78 ± 0.04^{a}	-	-	0.79 ± 0.04^{a}	-	-	-
Protocatechuic acid derivative I	4.90 ± 0.45^{a}	-	12.88 ± 0.61^{b}	-	-	-	-
Protocatechuic acid derivative II	0.23 ± 0.01^{a}	-	-	-	-	-	-
Quinic acid	7.81± 0.69^{a}	-	-	10.37 ± 0.19^{c}	11.21± 0.19^{b}	-	-
Gallic acid	0.53 ± 0.00^{b}	0.26 ± 0.01^{a}	-	-	1.34 ± 0.04^{d}	1.25 ± 0.01^{c}	-
p-hydroxybenzoic acid	0.44 ± 0.04^{a}	-	-	-	-	-	-
4-Hydroxybenzoic acid 4-O-glucoside	1.60 ± 0.14^{b}	-	-	-	-	-	t.r.
4-Hydroxybenzoic acid 4-O-glucoside isomer	1.20 ± 0.09^{a}	2.26 ± 0.11^{b}	-	-	t.r.	-	-
Eucomic acid	5.21 ± 0.31^{b}	2.69 ± 0.13^{a}	14.61 ± 0.56^{c}	-	-	-	-
Eucomic acid derivative I	1.26 ± 0.37^{a}	-	5.37 ± 0.53^{b}	-	-	-	-
Eucomic acid isomer I	0.49 ± 0.04a	-	-	2.76 ± 0.09^{d}	1.98 ± 0.07^{c}	-	1.46 ± 0.03^{b}
Eucomic acid isomer II	2.45 ± 0.24^{a}	-	-	-	-	-	-
p-coumaric acid	0.43 ± 0.07^{a}	-	-	-	-	-	-
p-coumaric acid derivative	0.52 ± 0.16^{a}	-	-	-	-	-	-
**Total Phenolic acids**	238.26 ± 11.91^{f}	273.42 ± 13.67^{g}	151.80 ± 7.59^{d}	90.20 ± 4.51^{c}	82.62 ± 4.31^{b}	182.21 ± 9.11^{e}	50.62 ± 2.53^{a}
**FLAVONOIDS**							
Quercetin glycoside(QG1) - Quercetin hexosyl pentosyl rhamnoside	1.02 ± 0.01^{c}	1.31 ± 0.07^{d}	-	9.11 ± 0.17^{e}	1.54 ± 0.03^{d}	0.69 ± 0.00^{b}	0.12 ± 0.00^{a}
Quercetin glycoside(QG2) - Quercetin hexose pentoside	0.11 ± 0.01^{a}	-	-	1.19 ± 0.06^{b}	-	-	-

Quercetin-3-O-rhamnosyl-rutinoside (QG3)	0.69 ± 0.03^{a}	-	-	-	-	-	-
Myricetin	n.d.	-	-	-	-	-	-
Myricitrin (myricetin 3-rhamnoside)	0.10 ± 0.04^{a}	-	-	-	-	-	-
Isorhamnetin glucoxyl-rhamnosyl-rhamnoside(IG1)	0.40 ± 0.02^{d}	0.46 ± 0.02^{e}	0.20 ± 0.01^{c}	0.42 ± 0.01^{d}	t.r.	0.10 ± 0.00^{b}	0.02 ± 0.00^{a}
Isorhamnetin glucoxyl-rhamnosyl-pentoside(IG2)	5.01 ± 0.0^{e}	5.62 ± 0.28^{f}	0.59 ± 0.03^{a}	1.45 ± 0.06^{b}	3.01 ± 0.15^{d}	2.22 ± 0.03^{c}	1.34 ± 0.00^{b}
**Total Flavonoids**	7.33 ± 0.3^{e}	7.39 ± 0.23^{e}	0.79 ± 0.04^{a}	12.17 ± 0.61^{d}	4.55 ± 0.23^{C}	3.01 ± 0.06^{b}	1.47 ± 0.07^{a}
**ELLAGIC ACIDS**							
Ellagic acid	2.44 ± 0.17^{b}	2.03 ± 0.10^{a}	-	2.54 ± 0.29^{a}	n.d.	2.10 ± 0.02^{a}	
Ellagic acid derivative I	3.36 ± 0.27^{a}	-	-	t.r.	-	-	-
Ellagic acid rhamnoside	t.r.	-	-	-		-	-
**Total Ellagic acids**	5.80 ± 0.29^{c}	2.03 ± 0.10^{b}	t.r.	2.54 ± 0.29^{a}	t.r.	2.10 ± 0.02^{b}	t.r.
**Total phenolic compounds**	251.38 ± 12.57^{f}	282.84 ± 14.14^{g}	152.60 ± 7.63^{d}	104.91 ± 5.24^{c}	87.17 ± 4.35^{b}	187.32 ± 9.37^{e}	52.09 ± 2.60^{a}

Results were expressed as mean ± standard deviation (n = 4). This came from obtaining at least two independent extracts (n = 2) and performing the determinations of each two times (n = 2). Superscript letters indicate statistically significant differences ($p \leq 0.05$) between tissues, products and by-products. 1 Expressed as mg/100g fresh weight. Abbreviations: n.d.: not detected. T.r.: trazes.

Table S3. Stability of the most abundant betalain and phenolic compounds in *Opuntia stricta var. Dillenii* fruit tissues (peel, pulp and whole fruit), jam production products (intermediate juice and jam) and by-product (bagasse) during *in vitro* gastro-intestinal static digestion

Compound	*in vitro* digestión phase	Fresh whole fruit (FWF)	Fresh Peel (PE)	Fresh Pulp (PU)	Bagasse By-product (BA)	Fresh pressed Juice (JU)	Jam (JA)
**BETALAINS**							
Betanin	Control	87.28 ± 0.96^{d}	83.12 ± 4.16^{Bc}	146.36 ± 2.08^{e}	11.21 ± 0.18^{a}	18.77 ± 0.02^{b}	-
	Oral	61.19 ± 3.11^{Bc}	126.09 ± 6.30^{Ce}	74.04 ± 3.84^{Bd}	3.62 ± 0.18^{Ba}	16.79 ± 0.31^{Bb}	-
	Gastric	65.60 ± 0.04^{Bc}	78.13 ± 1.40^{Bd}	83.88 ± 0.80^{Ce}	5.59 ± 0.09^{Ca}	18.09 ± 0.40^{Cb}	-
	Intestinal	19.57 ± 1.34^{Ac}	35.39 ± 2.79^{Ad}	33.59 ± 0.46^{Ad}	2.13 ± 0.16^{Aab}	5.36 ± 0.42^{Ab}	-
Isobetanin	Control	42.78 ± 0.04^{d}	25.31 ± 1.27^{Bc}	100.77 ± 1.48^{e}	9.78 ± 0.16^{a}	16.88 ± 0.01^{b}	-
	Oral	30.40 ± 1.58^{Bc}	42.09 ± 2.10^{Cd}	53.45 ± 2.81^{Be}	3.28 ± 0.18^{Ba}	15.36 ± 0.26^{Ab}	-
	Gastric	32.85 ± 0.08^{Bd}	26.35 ± 0.83^{Bc}	60.84 ± .071^{Be}	5.09 ± 0.11^{Ca}	16.41 ± 0.34^{Cb}	-
	Intestinal	9.78 ± 0.75^{Ac}	11.56 ± 0.42^{Ad}	23.79 ± 0.41^{Ae}	1.81 ± 0.15^{Aa}	4.46 ± 0.41^{Ab}	-
Betanidin	Control	1.15 ± 0.11^{Ba}	4.32 ± 0.22^{Da}	1.70 ± 0.09^{Ca}	0.24 ± 0.01^{Da}	0.45 ± 0.01^{Ba}	-
	Oral	1.78 ± 0.18^{Cd}	3.59 ± 0.28^{Ce}	1.44 ± 0.08^{Bc}	0.07 ± 0.00^{Ba}	0.49 ± 0.00^{Cb}	-
	Gastric	1.80 ± 0.06^{Cd}	2.00 ± 0.01^{Be}	1.56 ± 0.00^{BCc}	0.11 ± 0.00^{Ca}	0.50 ± 0.11^{Cb}	-
	Intestinal	0.54 ± 0.04^{Ac}	0.94 ± 0.04^{Ad}	0.62 ± 0.01^{Ac}	0.02 ± 0.00^{Aa}	0.12 ± 0.01^{Ab}	-
2´-O-apiosyl-4-O-phyllocactin	Control	45.86 ± 5.73^{Dc}	35.17 ± 1.76^{Cc}	110.06 ± 5.50^{Dd}	8.83 ± 0.08^{Db}	11.75 ± 0.40^{Ab}	0.51 ± 0.03^{Ca}
	Oral	29.52 ± 1.59^{Bc}	52.55 ± 2.63^{Dd}	51.73 ± 2.19^{Bd}	2.74 ± 0.05^{Ba}	12.16 ± 0.17^{Ab}	0.05 ± 0.01^{Aa}
	Gastric	29.30 ± 1.03^{Bd}	31.86 ± 0.17^{Be}	55.96 ± 0.45^{Cf}	3.04 ± 0.15^{Cb}	11.83 ± 0.07^{Ac}	0.08 ± 0.00^{Ba}
	Intestinal	9.25 ± 0.70^{Ac}	14.53 ± 0.27^{Ad}	6.60 ± 0.33^{Ab}	1.94 ± 0.05^{Aa}	n.d.	n.d.
Neobetanin	Control	17.17 ± 0.70^{Cc}	6.85 ± 0.34^{Ba}	56.57 ± 0.29^{Cd}	8.71 ± 0.19^{Db}	9.74 ± 0.27^{Cb}	-
	Oral	11.63 ± 1.13^{Bc}	8.01 ± 0.40^{Dbc}	35.02 ± 3.49^{Bd}	3.59 ± 0.22^{Ba}	10.76 ± 0.04^{Dc}	-
	Gastric	17.15 ± 0.97^{Cc}	7.69 ± 0.38^{Cb}	35.30 ± 2.33^{Bd}	4.24 ± 0.18^{Ca}	9.23 ± 0.06^{Bb}	-
	Intestinal	1.30 ± 0.30^{Aa}	1.59 ± 0.08^{Aa}	14.83 ± 1.56^{Ab}	0.27 ± 0.01^{Aa}	0.70 ± 0.03^{Aa}	-
	Control	194.23 ± 9.71^{De}	154.76 ± 7.74^{Bd}	415.46 ± 20.77^{Df}	38.76 ± 1.94^{Cb}	57.59 ± 2.88^{Cc}	0.51 ± 0.03^{Aa}
**Total of major**	Oral	134.53 ± 6.73^{Cd}	232.33 ± 11.62^{Cf}	215.69 ± 10.78^{Be}	13.29 ± 0.66^{Bb}	55.56 ± 2.78^{Bc}	0.05 ± 0.00^{Aa}

**betalains**	Gastric	100.66 ± 5.03^{Bd}	146.03 ± 7.30^{Be}	237.54 ± 11.88^{Cf}	18.07 ± 0.90^{Bb}	56.06 ± 2.80^{Bc}	0.08 ± 0.00^{Aa}
	Intestinal	40.44 ± 2.02^{Ad}	64.01 ± 3.20^{Ae}	79.43 ± 3.97^{Af}	6.18 ± 0.31^{Ab}	10.64 ± 0.53^{Ac}	0.00^{Aa}
**PHENOLICS ACIDS**							
Piscidic acid	Control	56.21 ± 0.30^{Cc}	101.00 ± 5.04^{Ce}	39.52 ± 0.65^{Ca}	47.85 ± 0.27^{Db}	60.74 ± 0.70^{Cd}	38.71 ± 2.13^{Ca}
	Oral	39.38 ± 1.75^{Bc}	113.04 ± 5.65^{Dd}	28.45 ± 0.24^{Ba}	21.24 ± 0.54^{Bb}	23.56 ± 0.46^{Ab}	28.97 ± 0.78^{ABa}
	Gastric	45.82 ± 4.64^{Bc}	76.02 ± 2.41^{Bc}	7.65 ± 0.42^{Ba}	25.40 ± 0.68^{Cb}	28.01 ± 0.21^{Bb}	12.85 ± 0.30^{Ba}
	Intestinal	34.35 ± 1.72^{ABd}	41.12 ± 2.81^{Ae}	2.93 ± 0.09^{Aab}	0.97 ± 0.03^{Aa}	26.83 ± 0.77^{Bc}	7.02 ± 1.10^{Ab}
**FLAVONOIDS**							
Quercetin	Control	1.02 ± 0.01^{Dc}	1.31 ± 0.07^{Ae}	-	1.54 ± 0.03^{Ad}	0.69 ± 0.00^{Cb}	0.12 ± 0.00^{Ba}
glycoside(QG1) -	Oral	1.08 ± 0.01^{Cb}	2.08 ± 0.40^{Bc}	-	n.d.	0.44 ± 0.00^{Ba}	0.01 ± 0.00^{Aa}
Quercetin hexosyl	Gastric	0.92 ± 0.00^{Bc}	1.13 ± 0.02^{Ad}	-	n.d.	0.47 ± 0.00^{Bb}	0.02 ± 0.00^{Aa}
pentosyl rhamnoside	Intestinal	0.29 ± 0.03^{Ab}	0.70 ± 0.07^{Ac}	-	n.d.	0.31 ± 0.01^{Ab}	0.01 ± 0.00^{Aa}
Isorhamnetin glucoxyl-	Control	0.40 ± 0.02^{Dd}	0.46 ± 0.02^{Be}	0.20 ± 0.01^{Ac}	-	0.10 ± 0.00^{Cb}	0.02 ± 0.00^{Ba}
rhamnosyl-	Oral	0.32 ± 0.02^{Cb}	0.77 ± 0.05^{Cc}	n.d.	-	0.07 ± 0.01^{Ba}	0.00 ± 0.00^{Aa}
rhamnoside(IG1)	Gastric	0.26 ± 0.02^{Bc}	0.37 ± 0.00^{Bd}	n.d.	-	0.10 ± 0.00^{Cb}	0.00 ± 0.00^{Aa}
	Intestinal	0.06 ± 0.00^{Aa}	0.14 ± 0.00^{Ab}	n.d.	-	0.03 ± 0.00^{Aa}	n.d.
Isorhamnetin glucoxyl-	Control	5.01 ± 0.03^{Ce}	5.62 ± 1.77^{Ae}	0.59 ± 0.03^{Ca}	3.01 ± 0.15^{Dd}	2.22 ± 0.03^{BCbc}	1.34 ± 0.00^{Bab}
rhamnosyl-	Oral	3.75 ± 0.37^{Bb}	9.68 ± 0.91^{Bc}	0.40 ± 0.08^{Ba}	1.07 ± 0.00^{Bab}	2.10 ± 0.11^{Bab}	0.01 ± 0.00^{Aa}
pentoside(IG2)	Gastric	3.74 ± 0.11^{Be}	5.96 ± 0.05^{Af}	0.41 ± 0.03^{Db}	1.69 ± 0.02^{Cc}	2.42 ± 0.04^{Cd}	0.02 ± 0.00^{Aa}
	Intestinal	1.00 ± 0.06^{Ae}	2.32 ± 0.06^{Af}	0.22 ± 0.03^{Ab}	0.56 ± 0.03^{Ac}	0.81 ± 0.01^{Ad}	0.01 ± 0.00^{Aa}
**Total of major**	Control	6.43 ± 0.32^{De}	7.38 ± 0.37^{Cf}	0.79 ± 0.04^{Aa}	4.55 ± 0.23^{Dd}	3.01 ± 0.15^{Cc}	1.47 ± 0.07^{Bab}
**Flavonoids**	Oral	5.15 ± 0.26^{Cd}	12.53 ± 0.63^{De}	0.40 ± 0.02^{Aa}	1.07 ± 0.05^{Bb}	2.61 ± 0.13^{Bc}	0.03 ± 0.00^{Aa}
	Gastric	4.92 ± 0.25^{Be}	7.19 ± 0.36^{Bf}	0.81 ± 0.04^{Ab}	1.69 ± 0.08^{Cc}	2.99 ± 0.15^{Cd}	0.04 ± 0.00^{Aa}
	Intestinal	1.35 ± 0.07^{Ae}	3.17 ± 0.16^{Af}	0.22 ± 0.01^{Ab}	0.56 ± 0.03^{Ac}	1.15 ± 0.06^{Ad}	0.02 ± 0.00^{Aa}
**Total of major**	Control	62.64 ± 3.13^{Dab}	108.39 ± 5.42^{Ce}	40.31 ± 2.02^{Da}	52.40 ± 2.62^{Dab}	63.76 ± 3.19^{Dab}	40.81 ± 2.01^{Da}
**phenolic compounds**	Oral	44.96 ± 2.25^{Bd}	125.57 ± 6.28^{De}	8.85 ± 0.44^{Cs}	22.30 ± 1.12^{Bb}	26.17 ± 1.31^{Ac}	8.99 ± 0.45^{Bd}

Gastric	50.73 ± 2.54^{Ce}	83.21 ± 4.16^{Bf}	8.45 ± 0.42^{Bs}	27.09 ± 1.35^{Cd}	31.00 ± 1.55^{Cc}	12.89 ± 0.64^{Cb}
Intestinal	35.70 ± 1.79^{Ae}	44.28 ± 2.21^{Af}	3.16 ± 0.16^{Ab}	1.53 ± 0.08^{Aa}	27.98 ± 1.40^{Bd}	7.04 ± 0.35^{Ac}

Results were expressed as mean ± standard deviation (n = 4). This came from obtaining at least two independent extracts (n = 2) and performing the determinations of each two times (n = 2). Superscript capital letters indicate statistically significant differences ($p \leq 0.05$) between digestion phases. Superscript small letters indicate statistically significant differences ($p \leq 0.05$) between tissues, products and by-products. 1 Expressed as mg/100g fresh weight. Abbreviations: n.d.: not detected.

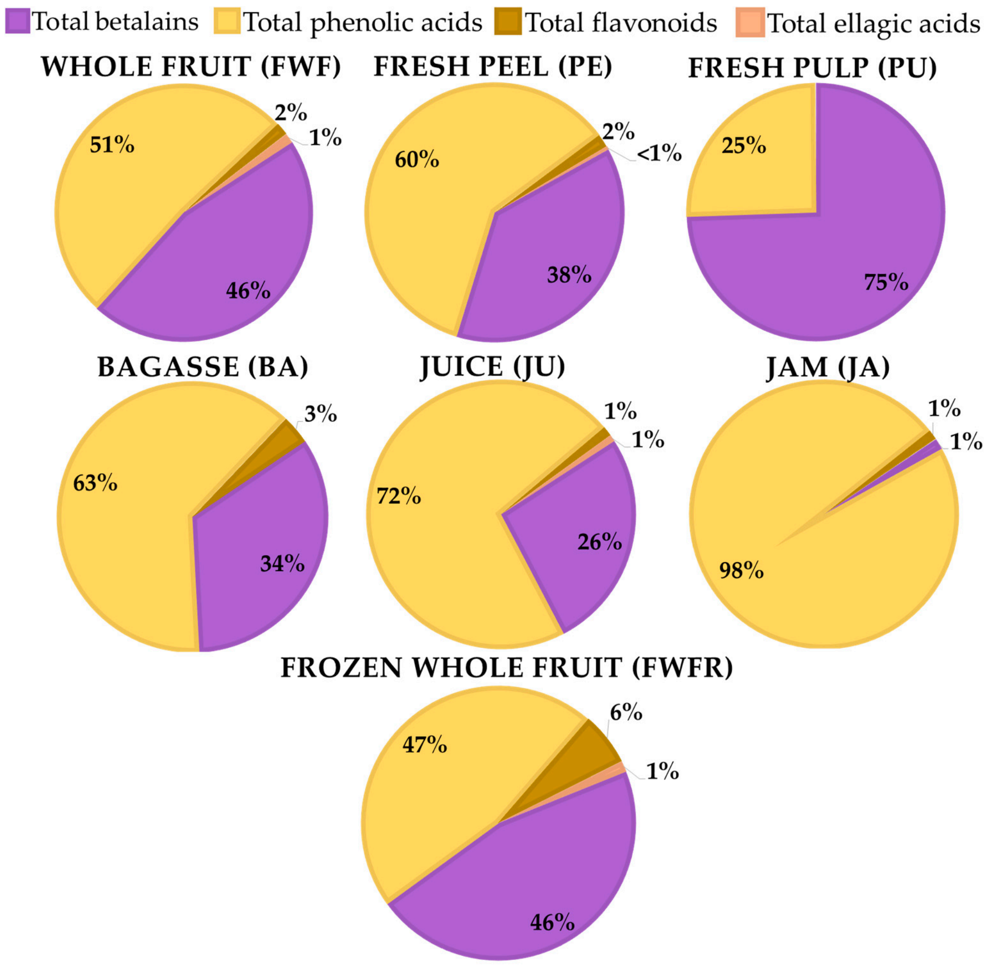

Figure S1. Betalains, phenolic acids and flavonoids distribution (%) from *Opuntia stricta var. Dillenii* fruit tissues (peel, pulp and whole fruit). jam production products (intermediate juice and jam) and by-product (bagasse).

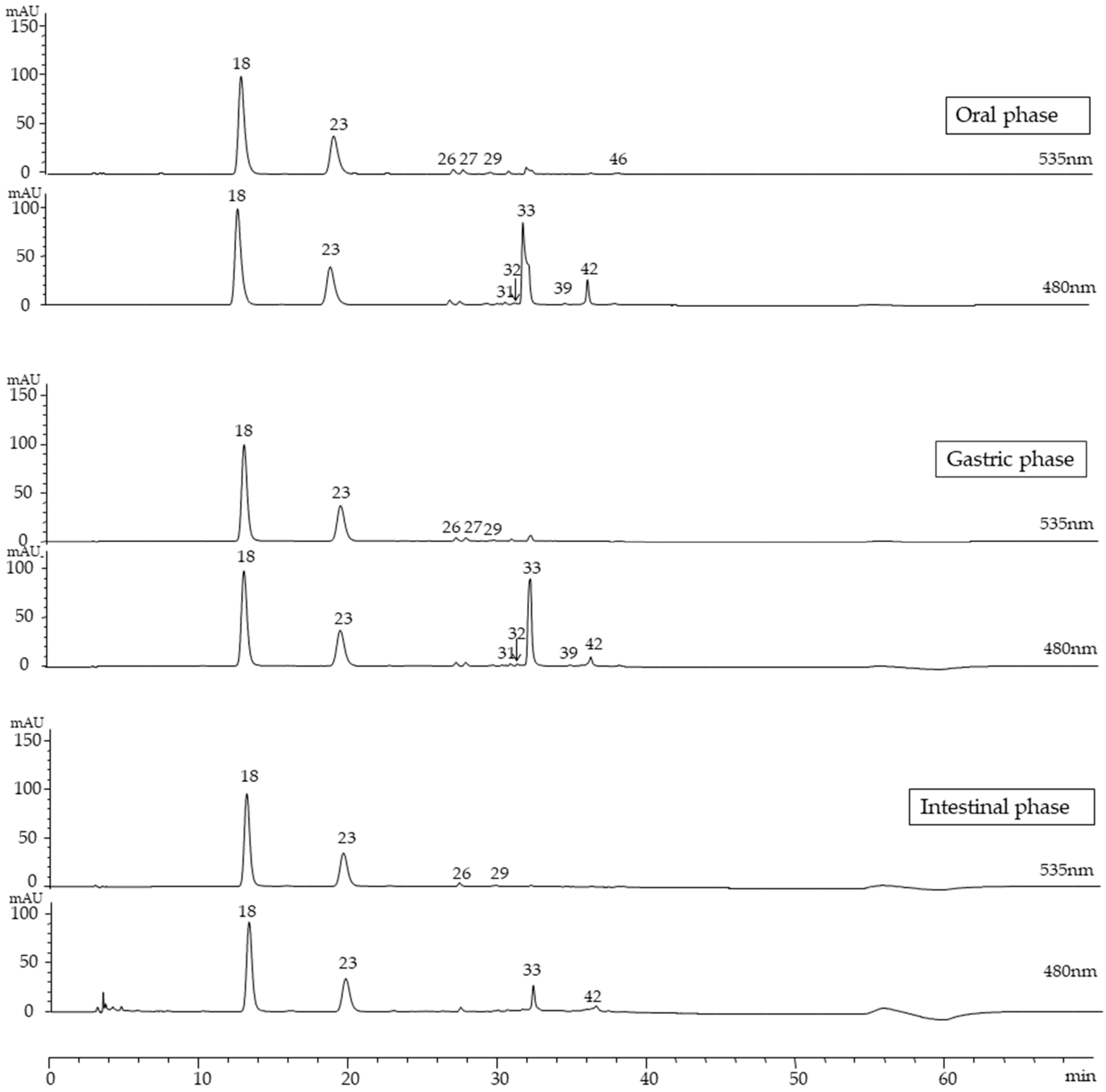

Figure S2. HPLC-DAD chromatograms of betalains compounds recorded at 535 and 480 nm, betacyanins and betaxanthins, respectively, obtained from *Opuntia stricta var. Dillenii* whole fruit after in vitro static gastro-intestinal digestion. [1]Numbers correspond to the identified compounds indicated in Table 1.

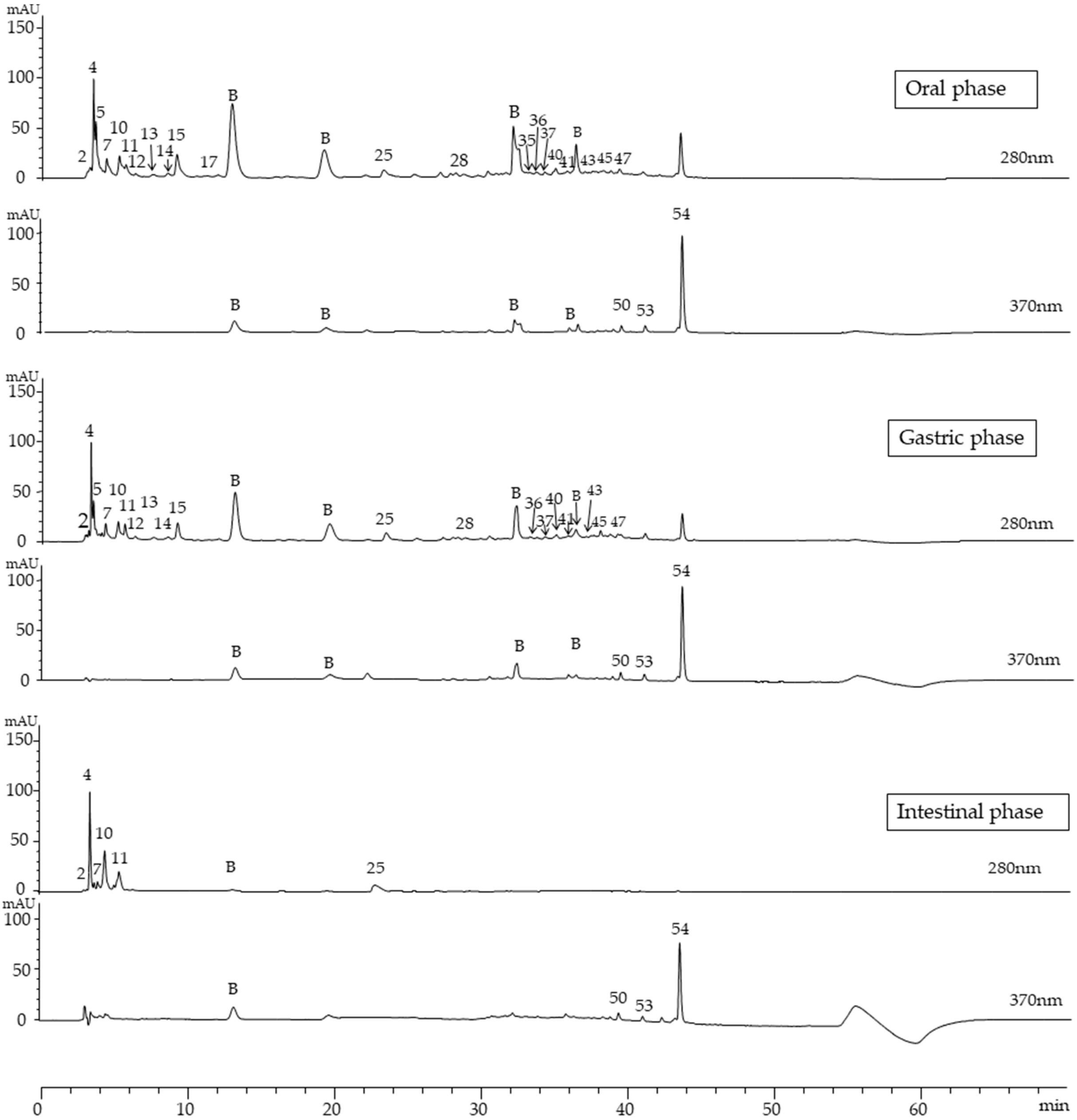

Figure S3. HPLC-DAD chromatograms of phenolic compounds recorded at 280 and 370 nm, phenolic acids and flavonoids, respectively, obtained from *Opuntia stricta var. Dillenii* whole fruit after in vitro static gastro-intestinal digestion. [1]Numbers correspond to the identified compounds indicated in Table 1.
